# Key Residues and Phosphate Release Routes in the *Saccharomyces cerevisiae* Pho84 Transceptor: THE ROLE OF TYR^179^ IN FUNCTIONAL REGULATION*[Fn FN2]

**DOI:** 10.1074/jbc.M116.738112

**Published:** 2016-11-08

**Authors:** Dieter R. Samyn, Jeroen Van der Veken, Griet Van Zeebroeck, Bengt L. Persson, Björn C. G. Karlsson

**Affiliations:** From the ‖ Computational Chemistry & Biochemistry Group,; the ‡ Linnæus University Centre for Biomaterials Chemistry, Linnæus University, SE-391 82 Kalmar, Sweden,; the § Department of Molecular Microbiology, VIB, Kasteelpark Arenberg 31, BE-3001 Leuven-Heverlee, Flanders, Belgium, and; the ¶ Laboratory of Molecular Cell Biology, Institute of Botany and Microbiology, Katholieke Universiteit Leuven, Kasteelpark Arenberg 31, BE-3001 Leuven-Heverlee, Flanders, Belgium

**Keywords:** cell signaling, membrane transport, molecular dynamics, Saccharomyces cerevisiae, yeast metabolism, Pho84, Phosphate transport, Transceptor

## Abstract

Pho84, a major facilitator superfamily (MFS) protein, is the main high-affinity P_i_ transceptor in *Saccharomyces cerevisiae*. Although transport mechanisms have been suggested for other MFS members, the key residues and molecular events driving transport by P_i_:H^+^ symporters are unclear. The current Pho84 transport model is based on the inward-facing occluded crystal structure of the Pho84 homologue PiPT in the fungus *Piriformospora indica*. However, this model is limited by the lack of experimental data on the regulatory residues for each stage of the transport cycle. In this study, an open, inward-facing conformation of Pho84 was used to study the release of P_i_. A comparison of this conformation with the model for P_i_ release in PiPT revealed that Tyr^179^ in Pho84 (Tyr^150^ in PiPT) is not part of the P_i_ binding site. This difference may be due to a lack of detailed information on the P_i_ release step in PiPT. Molecular dynamics simulations of Pho84 in which a residue adjacent to Tyr^179^, Asp^178^, is protonated revealed a conformational change in Pho84 from an open, inward-facing state to an occluded state. Tyr^179^ then became part of the binding site as was observed in the PiPT crystal structure. The importance of Tyr^179^ in regulating P_i_ release was supported by site-directed mutagenesis and transport assays. Using trehalase activity measurements, we demonstrated that the release of P_i_ is a critical step for transceptor signaling. Our results add to previous studies on PiPT, creating a more complete picture of the proton-coupled P_i_ transport cycle of a transceptor.

## Introduction

The major facilitator superfamily (MFS)^4^ is one of the largest families of secondary active transporters (1). Currently, 64 crystal structures from 10 different families are available, andseveral of these structures were resolved in substrate-free and substrate-bound conformational states, therefore providing significant insights into the molecular mechanisms involved in nutrient transport (2). The key feature of MFS transporters is that transport across the membrane occurs via an alternating-access mechanism, the “rocker-switch” model (3), which involves a variety of distinct conformations including outward-open, substrate-bound occluded, and inward-open states. A limitation of the rocker-switch model, however, is that because it is based solely on rigid body rotation, it does not explain the presence of the occluded state. To account for this limitation, the “clamp-and-switch” model has recently been proposed (2). This model suggests that the conformational transition process is more dynamic than previously assumed and that the switch between conformations is driven by pore-lining helices that bend to form the occluded state and that allow for outward-facing to inward-facing rotations and vice versa.

Inorganic phosphate (P_i_) is required for numerous cellular functions, such as synthesis of DNA and membrane lipids, intracellular signaling, and the generation of high-energy phosphate esters in ATP. Because of the central role of P_i_, cells have adopted strategies to ensure rapid cellular responses to internal and external fluctuations in P_i_ levels that may disturb cellular phosphate homeostasis. Moreover, several P_i_ transporters have been shown to transport P_i_ by H^+^-coupled symport (4).

Currently, the only available crystal structure of a P_i_:H^+^ transporter is the eukaryotic *Piriformospora indica* transporter PiPT, which was resolved in the substrate-bound inward-facing occluded conformation (5). The current model for P_i_:H^+^-transport is based on this crystal structure and describes three major conformational variations (*i.e.* substrate-docking, binding, and release) (5). A weakness of this model is that it lacks experimental data on the regulatory residues in each stage of the transport cycle.

The *Saccharomyces cerevisiae* Pho84 high-affinity phosphate transporter is a member of the phosphate:H^+^ symporter family (2.A.1.9) and is the main transporter during P_i_-limiting conditions. Furthermore, Pho84 activates the protein kinase A (PKA) pathway, which senses and signals the uptake of external P_i_ in phosphate-starved cells. This dual functionality of Pho84 has led to its classification as a transceptor (6). A three-dimensional model of Pho84 in the open inward-facing conformation has been created by homology modeling using the glycerol 3-phosphate transporter GlpT as a template (7). This model has been used to verify the roles of key residues in P_i_ binding, transport, and signaling (8). A comparison of the Pho84 and PiPT structures revealed a series of conserved residues in the P_i_ binding site (5). Interestingly, in contrast to Tyr^150^ in PiPT, Tyr^179^ in Pho84 is not in the binding site of the open inward-facing conformation. This difference may result from protonation/deprotonation of the nearby Asp^178^ residue (Asp^149^ in PiPT), which may affect the active conformation of the transporter. In addition, Asp^178^ has been shown to participate in H^+^-transfer (8). Nevertheless, the protonation/deprotonation mechanism of regulating conformational changes in P_i_:H^+^ transporters remains putative.

In this study, we examined the role of Tyr^179^ on the P_i_ release step in the open inward-facing conformation of Pho84. To address the finer details of the release mechanism and to build on the transport model previously suggested for PiPT, we performed a series of unrestrained molecular dynamics (MD) and steered molecular dynamics (SMD) simulations using different protonation states of P_i_ and Asp^178^.

These simulations revealed that the protonation state of Asp^178^ alters the conformational state of Pho84. Upon protonation of Asp^178^, Tyr^179^ underwent a rotameric change to become part of the binding site. This agrees with the contact found between P_i_ and Tyr^150^ in the PiPT occluded inward-facing conformation. Based on SMD simulations, different P_i_ release routes were suggested. The lowest-energy release route was found with H_2_PO_4_^−^ and deprotonated Asp^178^.

We also confirmed the importance of Tyr^179^ in regulating P_i_ transport by a series of site-directed mutagenesis studies and biochemical assays. Finally, we measured trehalase activity to determine whether Tyr^179^ regulated PKA signaling, and we demonstrated that the release of P_i_ is critical for signaling. Altogether, our data contributes to a more complete picture of the dual functions of phosphate transceptors.

## Results and Discussion

### 

#### 

##### Tyr^179^ Is Crucial for the Substrate Release Step of P_i_ Transport

**FIGURE 1.**
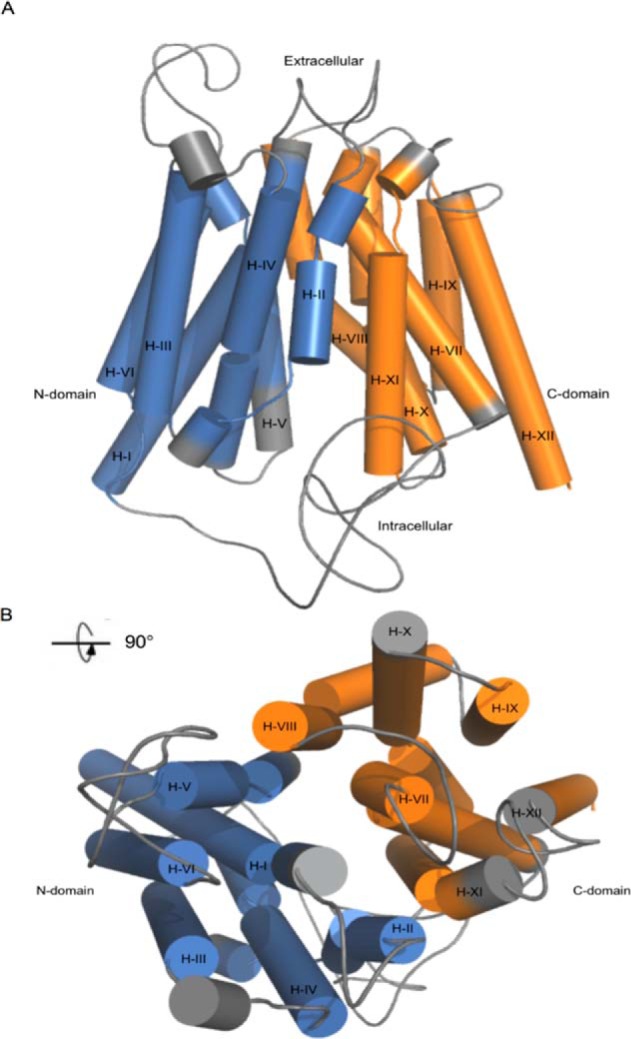
**The N-domain (transmembrane helices I-VI) and C-domain (transmembrane helices VII-XII) of Pho84 are shown in *blue* and *orange*, respectively.** *A,* frontal view of the Pho84 model. *B,* periplasmic views of the three-dimensional Pho84 model. All of the loops and non-transmembrane domains are shown in *gray*.

The two-dimensional topology of Pho84 consists of a C-domain and an N-domain, each domain is made up of six transmembrane segments (Table 1), and both the N and C termini are oriented toward the cytoplasm. The three-dimensional *in silico* model displays a Mayan temple shape (3) (Fig. 1*A*) with a distinct N- and C-terminal domain organization and a clearly visible transport channel in the center of the protein (Fig. 1*B*). The transport channel is a structural feature common in MFS proteins.

**TABLE 1:** **Amino acid sequences of transmembrane (TM) domains, loops (L), and the N and C termini according to Lagerstedt and co-workers (7)**

	Amino acid sequence (the position is given in superscript)
**Transmembrane domain**	
TM-I	Asp^55^-Glu-Gly-Phe-Gly-Trp-Gln-Gln-Val-Lys-Thr-Ile-Ser-Ile-Ala-Gly-Val-Gly-Phe-Leu-Thr-Asp-Ser-Tyr-Asp-Ile-Phe-Ala-Ile-Asn-Leu-Gly-Ile^87^
TM-II	Gln^104^-Thr-Leu-Leu-Lys-Val-Ser-Thr-Ser-Val-Gly-Thr-Val-Ile-Gly-Gln-Phe-Gly-Phe-Gly-Thr-Leu-Ala^126^
TM-III	Gly^130^-Arg-Lys-Arg-Ile-Tyr-Gly-Met-Glu-Leu-Ile-Ile-Met-Ile-Val-Cys-Thr-Ile-Leu-Gln-Thr-Thr^151^
TM-IV	Val^163^-Leu-Thr-Phe-Tyr-Arg-Ile-Val-Met-Gly-Ile-Gly-Ile-Gly-Gly-Asp-Tyr-Pro-Leu-Ser-Ser-Ile-Ile-Thr^186^
TM-V	Ala^201^-Val-Phe-Ala-Asn-Gln-Ala-Trp-Gly-Gln-Ile-Ser-Gly-Gly-Ile-Ile-Ala-Leu-Ile-Leu^220^
TM-VI	Gly^254^-Leu-Gly-Thr-Val-Leu-Gly-Leu-Ala-Cys-Leu-Tyr-Phe-Arg-Leu-Thr-Ile-Pro-Glu-Ser-Pro-Arg-Tyr-Gln-Leu^278^
TM-VII	Leu^347^-Leu-Gly-Thr-Ala-Gly-Ser-Trp-Phe-Thr-Leu-Asp-Val-Ala-Phe-Tyr-Gly-Leu-Ser-Leu-Asn-Ser-Ala-Val-Ile^371^
TM-VIII	Tyr^388^-Asp-Thr-Ala-Val-Gly-Asn-Leu-Ile-Leu-Ile-Cys-Ala-Gly-Ser-Leu-Pro-Gly-Tyr-Trp-Val-Ser-Val-Phe-Thr-Val-Asp-Ile-Ile^416^
TM-IX	Gln^422^-Leu-Ala-Gly-Phe-Ile-Ile-Leu-Thr-Ala-Leu-Phe-Cys-Val-Ile-Gly-Phe-Ala-Tyr^440^
TM-X	Gly^447^-Leu-Leu-Ala-Leu-Tyr-Val-Ile-Cys-Gln-Phe-Phe-Gln-Asn-Phe-Gly-Pro-Asn-Thr-Thr-Thr-Phe-Ile-Val-Pro-Gly-Glu^473^
TM-XI	Tyr^479^-Arg-Ser-Thr-Ala-His-Gly-Ile-Ser-Ala-Ala-Ser-Gly-Lys-Val-Gly-Ala-Ile-Ile-Ala-Gln-Thr-Ala-Leu-Gly-Thr^504^
TM-XII	Met^525^-Glu-Ile-Phe-Ala-Leu-Phe-Met-Leu-Leu-Gly-Ile-Phe-Thr-Thr-Leu-Leu-Ile-Pro-Glu-Thr-Lys-Arg-Lys-Thr-Leu-Glu^551^

**Loop**	
L-I	Thr^88^-Met-Met-Ser-Tyr-Val-Tyr-Trp-His-Gly-Ser-Met-Pro-Gly-Pro-Ser^103^
L-II	Asp^127^-Ile-Val^129^
L-III	Val^152^-Ala-His-Ser-Pro-Ala-Ile-Asn-Phe-Val-Ala^162^
L-IV	Ser^187^-Glu-Phe-Ala-Thr-Thr-Lys-Trp-Arg-Gly-Ala-Ile-Met-Gly^200^
L-V	Val^221^-Ala-Ala-Tyr-Lys-Gly-Glu-Leu-Glu-Tyr-Ala-Asn-Ser-Gly-Ala-Glu-Cys-Asp-Ala-Arg-Cys-Gln-Lys-Ala-Cys-Asp-Gln-Met-Trp-Arg-Ile-Leu-Ile^253^
L-VI	Asp^279^-Val-Asn-Ala-Lys-Leu-Glu-Leu-Ala-Ala-Ala-Ala-Gln-Glu-Gln-Asp-Gly-Glu-Lys-Lys-Ile-His-Asp-Thr-Ser-Asp-Glu-Asp-Met-Ala-Ile-Asn-Gly-Leu-Glu-Arg-Ala-Ser-Thr-Ala-Val-Glu-Ser-Leu-Asp-Asn-His-Pro-Pro-Lys-Ala-Ser-Phe-Lys-Asp-Phe-Cys-Arg-His-Phe-Gly-Gln-Trp-Lys-Tyr-Gly-Lys-Ile^346^
L-VII	Leu^372^-Gln-Thr-Ile-Gly-Tyr-Ala-Gly-Ser-Lys-Asn-Val-Tyr-Lys-Lys-Leu^387^
L-VIII	Gly^417^-Arg-Lys-Pro-Ile^421^
L-IX	His^441^-Lys-Leu-Gly-Asp-His^446^
L-X	Cys^474^-Phe-Pro-Thr-Arg^478^
L-XI	Leu^505^-Ile-Asp-His-Asn-Cys-Ala-Arg-Asp-Gly-Lys-Pro-Thr-Asn-Cys-Trp-Leu-Pro-His-Val^524^

**Termini**	
N-terminal	Met^1^-Ser-Ser-Val-Asn-Lys-Asp-Thr-Ile-His-Val-Ala-Glu-Arg-Ser-Leu-His-Lys-Glu-His-Leu-Thr-Glu-Gly-Gly-Asn-Met-Ala-Phe-His-Asn-His-Leu-Asn-Asp-Phe-Ala-His-Ile-Glu-Asp-Pro-Leu-Glu-Arg-Arg-Arg-Leu-Ala-Leu-Glu-Ser-Ile-Asp^54^
C-terminal	Glu^552^-Ile-Asn-Glu-Leu-Tyr-His-Asp-Glu-Ile-Asp-Pro-Ala-Thr-Leu-Asn-Phe-Arg-Asn-Lys-Asn-Asn-Asp-Ile-Glu-Ser-Ser-Ser-Pro-Ser-Gln-Leu-Gln-His-Glu-Ala^587^

**FIGURE 2.**
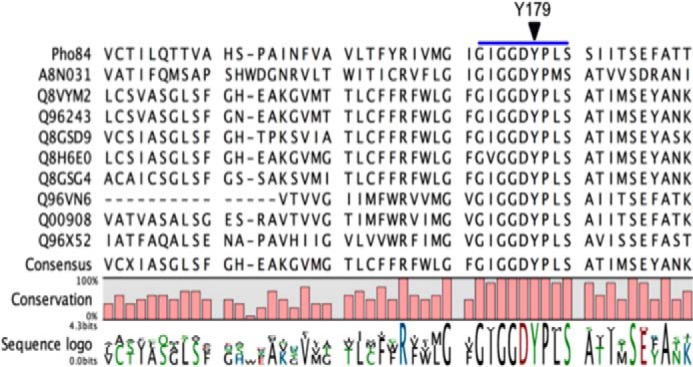
**A section of a multiple amino acid sequence alignment of the *S. cerevisiae* Pho84 protein (PM0076296) with homologues from *P. indica* (A8N031), *A. thaliana* Pht1–1 (Q8VYM2), *A. thaliana* Pht 1–2 (Q96243), *O. sativa* (Q8GSD9), *H. vulgare* (Q8H6E0), *M. truncatula* (Q8GSG4), *G. intraradices* (Q96VN6), *G. versiforme* (Q00908), and *P. nameko* (Q96X52).** The *blue line* indicates the part of the sequence motif that is shared among proton-coupled phosphate transporters in plants, fungi, bacteria, and mammals (TLCFFRFWLGFGIGGDYPLSATIMSE) (43). This signature sequence contains the phosphate binding sequence G*X*G*X*GG. The residue Tyr^179^ of the Pho84 transporter is indicated by an *inverted triangle*. Created with CLC workbench 7 (Qiagen, Aarhus, Denmark).

**FIGURE 3.**
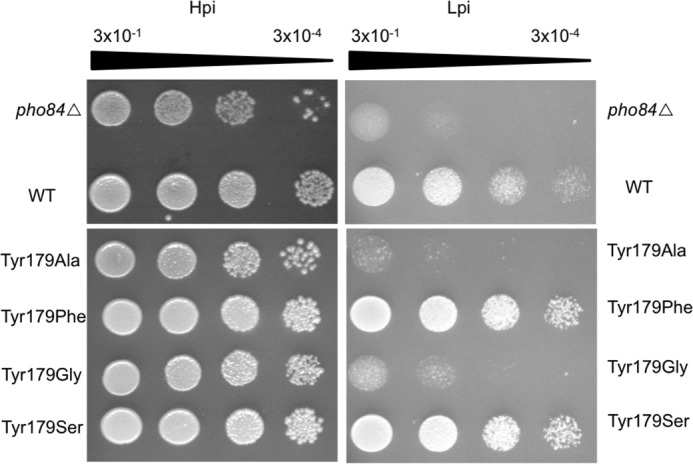
**Yeast growth spot test images were taken after 2 days of growth under HP_i_ conditions and after 3 days of growth under LP_i_ conditions.** To visualize the reduced growth of the *pho84*Δ and mutant strains under LP_i_ conditions, images were acquired with a higher illumination setting resulting in a brighter background. All of the strains were dilution-plated as indicated.

**FIGURE 4.**
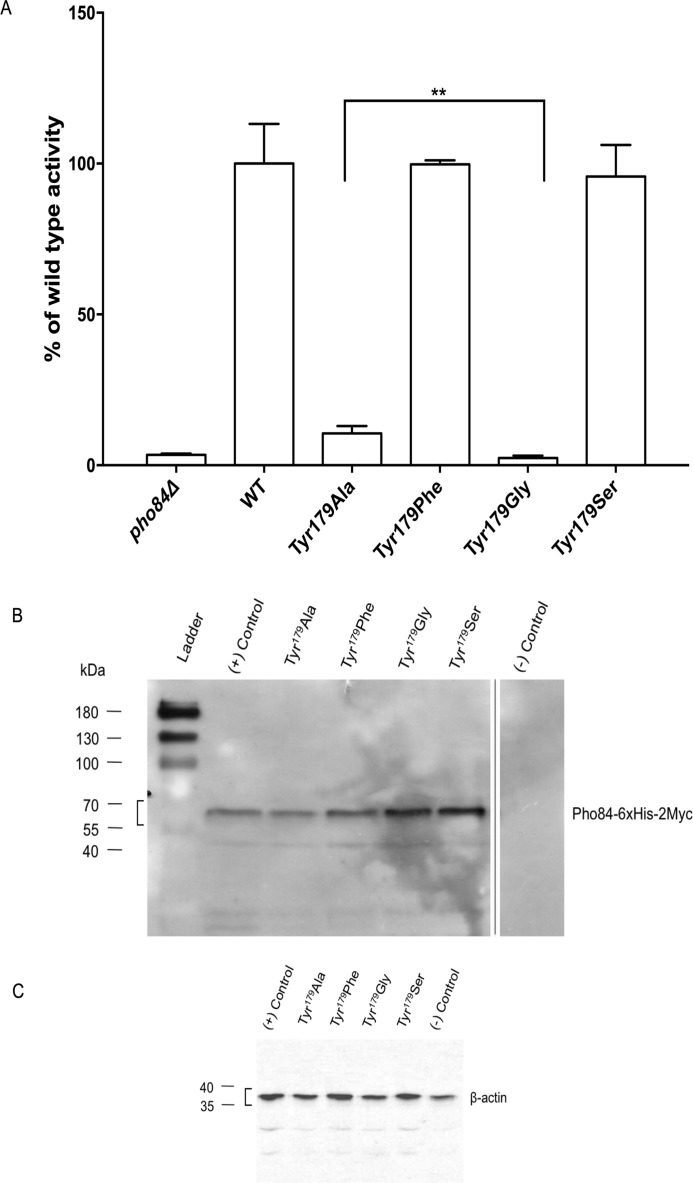
*A*, total transport activity was measured using radioactive phosphate in a short-term uptake assay with a saturating phosphate concentration (110 μm KH_2_PO_4_). Cells were grown in LP_i_ conditions. Experiments were performed in triplicate. Data are expressed as percentages of WT activity (14.658 nmol min^−1^ mg^−1^ cells, dry weight set as 100%), mean ± S.D. **, *p* < 0.05, significantly different (Student's *t* test). *B,* immunoblot with anti-c-Myc antibody to detect membrane enrichment. CEN.PK 113 7D strain was used as the (+) control, the CEN.PK 5D *PHO84*Δ was used as the (−) control. The band for the recombinant Pho84–6xHis-2Myc (apparent molecular weight of 66 kDa) is indicated with a *square bracket. C,* loading controls using complete yeast extract (10 μg) and anti-β-actin. The band for β-actin is indicated with a *square bracket*.

A multiple sequence alignment (MSA) analysis of Pho84 with other P_i_:H^+^ transporters revealed that Tyr^179^ is highly conserved (Fig. 2). Furthermore, the corresponding Tyr^150^ is proposed to be in the P_i_ binding site of the PiPT crystal structure. In the inward-open conformation of the Pho84 model, Tyr^179^ orients toward the cytoplasm and points away from the binding cavity. A growth spot test with strains expressing mutant alleles of *PHO84* was performed to assess changes in the transport activity of Pho84 (Fig. 3). When compared with the wild-type strain (CEN.PK 113–7D *PHO84*-6xHis-2xmyc), the strains expressing Pho84 Tyr^179^-Ala and Tyr^179^-Gly exhibited drastic reductions in growth in low-P_i_ (LP_i_) conditions indicating that Tyr^179^ is crucial for transport. The growth of the strains expressing Pho84 Tyr^179^-Ser and Tyr^179^-Phe was equivalent to the growth of the Pho84 wild-type strain. In addition, *in vivo* radiolabeled P_i_ uptake assays showed a drastic reduction in transport activity in the Tyr^179^-Ala mutant and abolished activity in the Tyr^179^-Gly mutant (Fig. 4*A*). The Tyr^179^-Ser and Tyr^179^-Phe mutant strains had uptake activities equivalent to the uptake activity in the Pho84 wild-type strain. Immunoblot analysis confirmed that the site-directed mutations yielded full-length recombinant proteins (Fig. 4*B*).

**FIGURE 5.**
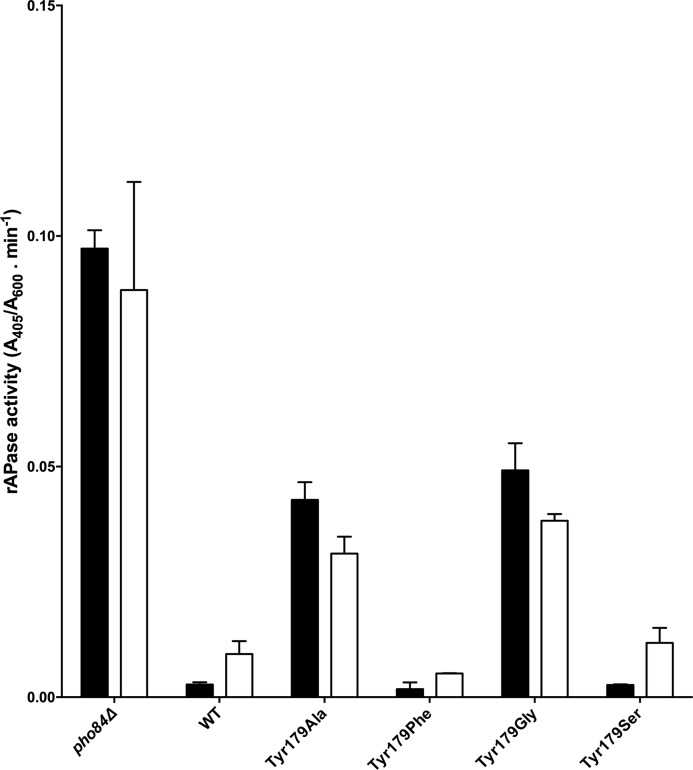
**Acid phosphatase (rAPase) activity assays of strains expressing mutant forms of Pho84 grown under HP_i_ (*closed bars*) and LP_i_ (*open bars*) conditions.** All measurements were performed in duplicate with three technical repetitions each and the results are shown as mean ± S.D.

Furthermore, expression of a functional Pho84 transporter is required to suppress the repressible acid phosphatase (rAPase) activity of *PHO5*, the major acidic phosphatase (9). A loss-of-function strain, *pho84*Δ, results in constitutive expression of *PHO5* independent of the external P_i_ conditions (Fig. 5). The wild-type strain was analyzed under either high-P_i_ (HP_i_) or low-P_i_ (LP_i_) conditions (8) revealing reduced and elevated secreted phosphatase activity, respectively. Strains expressing Pho84 Tyr^179^-Ala and Tyr^179^-Gly exhibited pronounced increases in secreted phosphatase activity, which is consistent with the significantly reduced transport activities of these mutants. Strains expressing Pho84 Tyr^179^-Ser and Tyr^179^-Phe had secreted phosphatase activities similar to the wild-type level. These results show that full-length expressed, plasma membrane localized, non-functional Pho84 suppresses rAPase activity resulting in less activity relative to the activity in *pho84*Δ (9).

Our data for Tyr^179^ clearly shows that this residue plays a crucial role in P_i_ transport. By introducing a side chain that is relatively inert, such as a residue that is non-charged, weakly polar, or lacking an aromatic residue, transport activity was largely abolished. In contrast, by introducing side chains with increased polarity (Tyr^179^-Ser) or hydrophobicity (Tyr^179^-Phe), transport activity was restored to the wild-type level.

##### Protonation/Deprotonation of Asp^178^ and Its Impact on P_i_ Release

A protonation/deprotonation cycle has been shown to be crucial for the functionality of many transporters. The *Escherichia coli* lactose permease LacY serves as a paradigm for understanding the importance of protonation/deprotonation in the transport cycle that begins in the outward-open conformation and requires functional protonation to facilitate substrate binding (10). MD simulations of the protonation/deprotonation of Glu^325^ showed significant changes in the structure leading to a transition from the inward-facing to the occluded conformation (11). MD simulations of the *E. coli* sugar fucose:H^+^ symporter FucP also showed that protonation of Glu^135^ is required to trigger conversion from the outward-open to the inward-open conformation (12). These examples show that protonation/deprotonation events are crucial to the alternating-access dynamics of MFS transporters.

In a previous site-directed mutagenesis study, the Pho84 residue Asp^178^ was mutated to Glu and Asn, and Asn was found to mimic a protonated aspartate (8). These results led to the hypothesis that protonation of Asp^178^ plays a role in a late phosphate release step of the transport cycle. To investigate this hypothesis, a series of unrestrained MD simulations were performed on Pho84 models with protonated or deprotonated Asp^178^. P_i_ was inserted in the Pho84 binding site at approximately equal distances from Lys^492^ and Asp^178^. This allowed us to investigate the roles of these residues on P_i_ binding and on proton transfer. In addition, simulations were conducted using different protonation states of P_i_ (H_3_PO_4_, H_2_PO_4_^−^, and HPO_4_^2−^) to resolve the role of charge balance in the binding site and its influence on the energetics of cytosolic release of P_i_ (see supplemental Table S1 for information on the number of molecules in each system and the denotations that are used throughout this paper). All of the systems were subsequently subjected to 30 ns of equilibration to reach stable energy, temperature, and density values (supplemental Figs. S1–S16). Equilibration of the transmembrane helices (Fig. 1) in the 1-palmitoyl-2-oleoyl-*sn*-glycero-3-phosphocholine bilayer were verified by recording root mean square deviation values over time for backbone (N, Cα, and C) atoms. These values reached a plateau after ∼15 ns with values of 3.5 Å or less (supplemental Fig. S17).

**FIGURE 6.**
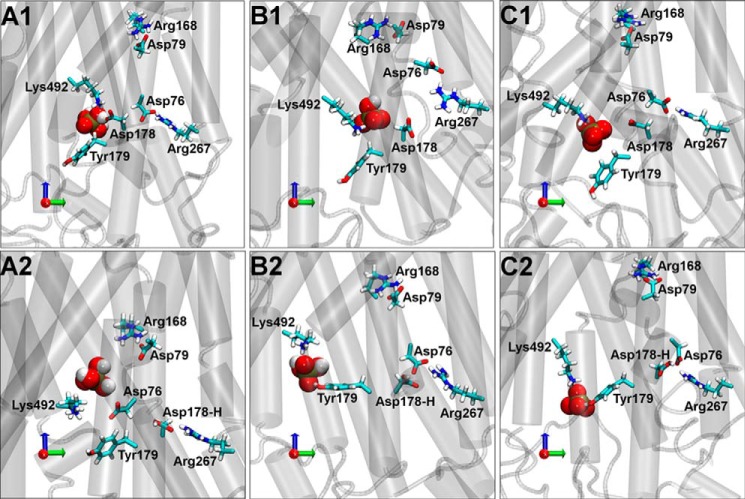
**Snapshots from simulations of various protonation states of P_i_ and Asp^178^ after 30 ns of unrestrained equilibration.** *A1*, H_3_PO_4_; *A2*, H_3_PO_4_ and protonated Asp^178^; *B1*, H_2_PO_4_^−^; *B2*, H_2_PO_4_^−^ and protonated Asp^178^; *C1*, HPO_4_^2−^; *C2*, HPO_4_^2−^ and protonated Asp^178^. Selected residues are shown. Lys^492^ and Tyr^179^ are proposed to be involved in P_i_ binding (van der Waals representation). Asp^178^, Asp^76^, and Asp^79^ are proposed to be involved in proton shuffling. Arg^267^ is proposed to form a salt bridge with Asp^76^, and Arg^168^ is proposed to form a salt bridge with Asp^79^.

Extracted snapshots of the Pho84 binding site after 30 ns of equilibration revealed the position of P_i_ in the binding site, the location of residues important for P_i_ binding, and the location of proton transfer (Fig. 6). Analysis of the position of H_3_PO_4_ and its variations in distance over time (Fig. 6, *A1*, and supplemental Fig. S18) revealed stable interactions between H_3_PO_4_, Lys^492^, and Asp^178^. Models using H_2_PO_4_^−^ or HPO_4_^2−^ revealed weakened interactions with Asp^178^ due to repulsion between mono- and divalent species of P_i_ and the deprotonated state of Asp^178^ (Fig. 6, *B1* and *C1*, and supplemental Fig. S18). Moreover, systems with either H_2_PO_4_^−^ or HPO_4_^2−^ in the binding site and a protonated state of Asp^178^ (Fig. 6, *B2* and *C2*, supplemental Figs. S18 and S19) revealed the formation of a hydrogen bond between Asp^178^ and the adjacent Asp^76^ residue resulting in less distance between helix I that harbors Asp^76^ and helix IV that harbors Asp^178^. Similar interactions were seen in the absence of P_i_ indicating that the proton is not donated by P_i_ (supplemental Fig. S19). These data confirm the role of Asp^178^ in proton-coupling (8). In addition, it has been suggested that the symport of protons through Pho84 occurs at a P_i_:H^+^ stoichiometry of 1:3 (13).

The largest shift in the distance between Asp^178^ and Asp^76^ upon protonation of Asp^178^ was observed for H_2_PO_4_^−^ rather than for HPO_4_^2−^ suggesting that H_2_PO_4_^−^ has the largest impact on the conformational flexibility of Pho84. Simulations with H_3_PO_4_ in the binding site and protonated Asp^178^ resulted in a helical rotation that affected helices I and IV such that Asp^76^, rather than Asp^178^, bound to P_i_ (Fig. 6, *A2*, and supplemental Fig. S18). These data demonstrate that the charge distribution in the Pho84 binding site regulates the active conformational state of Pho84.

In addition to the observed roles of Asp^178^ in regulating P_i_ binding and proton-coupling, the change in the conformational state of helix IV in Pho84 that occurred upon protonation of Asp^178^ led to Tyr^179^ becoming more accessible for P_i_ binding (Fig. 6, *B2* and *C2*, and supplemental Fig. S20). X-ray crystallographic analysis of PiPT in the substrate-bound inward-facing occluded conformation shows that the corresponding tyrosine (Tyr^150^) is involved in P_i_ binding (5). As shown in a comparison of Fig. 6, *panels A1*/*A2 versus panels B1*/*B2*/*C1*/*C2*, the shift in the position of Tyr^179^ as correlated to the protonation state of Asp^178^ was not as prominent for models that incorporated H_3_PO_4_.

We investigated the stability of salt bridges involving Asp^76^ and Asp^79^ and their link to Pho84 conformational changes (Fig. 6). We found stable contacts between Asp^76^-Arg^267^ and Asp^79^-Arg^168^ in all of the simulations with the exception of the model incorporating H_3_PO_4_ and protonated Asp^178^ in which Asp^178^ and Asp^76^ exchanged positions (Fig. 6, *A2*). Moreover, a Pho84 simulation without P_i_ in the binding site showed a disruption of the Asp^79^-Arg^168^ salt bridge after 15 ns of equilibration. Because protonation of Asp^178^ led to a stable Asp^79^-Arg^168^ salt bridge, we suggest that the presence of P_i_ in the binding site may increase the conformational stability of Pho84 (data not shown).

##### Characterizing Alternative Release Routes Using SMD Simulations

A series of SMD simulations were performed to investigate alternative P_i_ release routes in Pho84. For each model investigated, five separate SMD simulations were performed to sample the configurational space provided by the flexible Lys^492^ residue and to identify possible Pho84 exit routes using various protonation states of both Asp^178^ and P_i_. Although, according to the Jarzynski equality method that requires a large number of samplings to be performed (14–16), five simulations for each system is not enough to estimate the free energies of the possible release pathways, we used the simulations to qualitatively, rather than quantitatively, describe the alternative release routes and their corresponding forces and energies.

**FIGURE 7.**
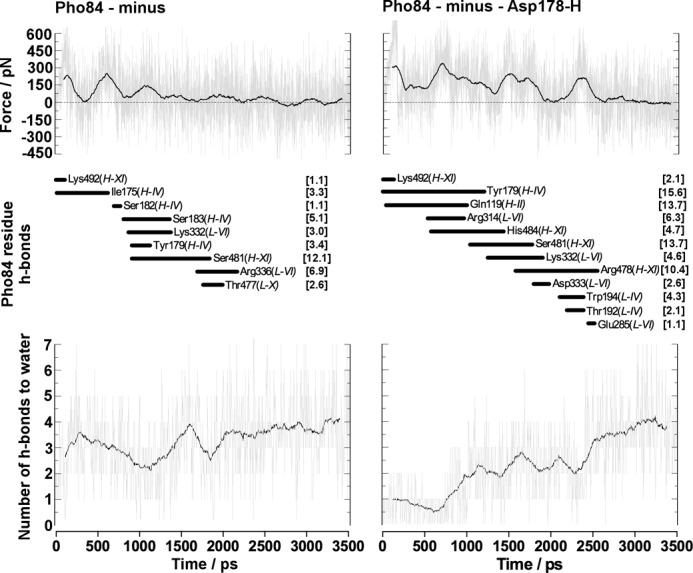
**Representative data from steered dynamics simulations for the release of P_i_ from the Pho84 binding site after initially restraining the Lys^492^-P_i_ distance for 0.1 ns.** The calculated force profiles and the Pho84 residues (and their transmembrane helix) that form hydrogen bonds with P_i_ along the release pathway or the average number of hydrogen bonds formed with water along the route for models involving deprotonated and protonated Asp^178^. *Numbers in brackets* represent hydrogen bonding between the residue and H_2_PO_4_^−^ as a percentage of the total simulation time.

Calculation of force profiles (supplemental Figs. S21–S22), work (supplemental Figs. S23–S24), as well as hydrogen bond contacts between residues and P_i_ (supplemental Tables S2–S5 and Figs. S25–S28) and water molecules and P_i_ (supplemental Fig. S29 –S30) along the series of routes populated allowed for a deeper understanding of the molecular basis of the regulation of P_i_ release in Pho84. The steered MD simulations suggested that the lowest-energy release route involved H_2_PO_4_^−^ and deprotonated Asp^178^ (supplemental Figs. S23–S34). SMD (Fig. 7) and unrestrained simulations demonstrated that protonation of Asp^178^ resulted in a rotation of helix IV, which increased the work required to release P_i_ via this route. The increased work required could be a result of the increase in hydrogen bond contacts and the number of water molecules encountered by H_2_PO_4_^−^ when Asp^178^ is protonated. A 30-ns unrestrained simulation showed that rotation of Tyr^179^ into the binding site made it more accessible for P_i_ binding. This increased binding between P_i_ and Tyr^179^ upon protonation of Asp^178^ indicated that Pho84 regulates its conformational state preceding P_i_ release. The lowest-energy route for the release of H_2_PO_4_^−^ from Pho84 involves helices IV and XI and a minimum number of contacts with residues in L-VI. Upon protonation of Asp^178^, however, the transport channel of Pho84 narrowed as a result of increased contacts with Gln^119^ in helix II and increased contacts with L-VI residues. In this protonated Pho84, H_2_PO_4_^−^ must be released via a higher energy route (Fig. 7). A detailed analysis of the number of hydrogen bond contacts formed between H_2_PO_4_^−^ and water along the release route (Fig. 7) demonstrated a reduction in hydrogen bonding between P_i_ and water. This reduction resulted from increased hydrogen bonding to Tyr^179^ in the binding site upon protonation of Asp^178^; these data support the observation that the release route narrowed. In addition, H_3_PO_4_ was found to contact Gln^119^ in helix II and to make contacts with L-VI residues suggesting that this alternate high-energy release route is used for H_3_PO_4_ as well as for H_2_PO_4_^−^. SMD simulations of the cytosolic release of HPO_4_^2−^ showed that this protonation state of P_i_ binds more strongly to Pho84 than any of the other investigated protonation states, thus charge balance in the transport channel appears to regulate P_i_ release routes (supplemental Figs. S22, S28, S30, and S34 and Table S5).

##### The Proposed Mechanism for P_i_ Release in P_i_:H^+^ Transporters Using Pho84 as a Model

Results from unrestrained and steered MD simulations showed that the protonation states of P_i_ and Asp^178^ and the resulting orientations of Tyr^179^ impact regulation of the P_i_ release pathway. Protonation of Asp^178^ is a crucial step in triggering the conformational change of Pho84 from an open inward-facing conformation to a more occluded conformation. However, this switch in conformation appears to be independent of the protonation state of P_i_. As a result of Pho84 adopting the occluded conformation, Tyr^179^ becomes part of the binding site as seen in the PiPT crystal structure.

**FIGURE 8.**
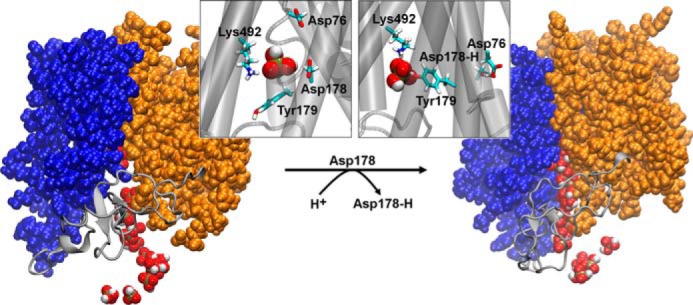
**A proposed mechanism for the release step in P_i_:H^+^ transporters using Pho84 as a model system.** Snapshots representing the inward-facing open (image on the *left*) and occluded (image on the *right*) conformational states of Pho84 after simulating protonated (Asp^178^-H) or deprotonated states (Asp^178^) of Asp^178^. Detailed views of the binding site (helices are represented as *gray cylinders*) in both conformational states of Pho84 clearly show H_2_PO_4_^−^ in the binding site and residue Tyr^179^ either oriented away from the binding site (Asp^178^) or toward the binding site (Asp^178^-H). The structures shown were obtained after 3.5 ns of steered MD simulations and multiple superimposed structures of P_i_ (van der Waals representation, extracted every 0.175 ns). Moreover, the N (transmembrane helices I-VI)- and C (transmembrane helices VII-XII)-domains of Pho84 are shown as *blue* and *orange* van der Waals representations, respectively. The connecting loop between these domains, L-VI, is shown as a *gray schematic* representation.

Based on our observations, we propose a sequential mechanism for the release of P_i_ (Fig. 8).

(i) The open inward-facing conformation of Pho84 containing deprotonated Asp^178^ is the active form that releases P_i_. We found that H_2_PO_4_^−^ is the most favorable protonation state of P_i_ to be released due to the distribution of the charges of the residues along the release pathway. In this active conformation of Pho84, Tyr^179^ points away from the binding site and may regulate the possible H_2_PO_4_^−^ release routes and/or may act as a gatekeeper, which blocks more hydrophobic phosphate substrates.

(ii) Protonation of Asp^178^ results in a conformational change from an open inward-facing conformation to an occluded conformation in which Tyr^179^ is reoriented into the binding site and Pho84 is locked into a closed and inactive conformation, which is unable to release H_2_PO_4_^−^.

##### Transceptor Function Is Coupled to the Transport Cycle

**FIGURE 9.**
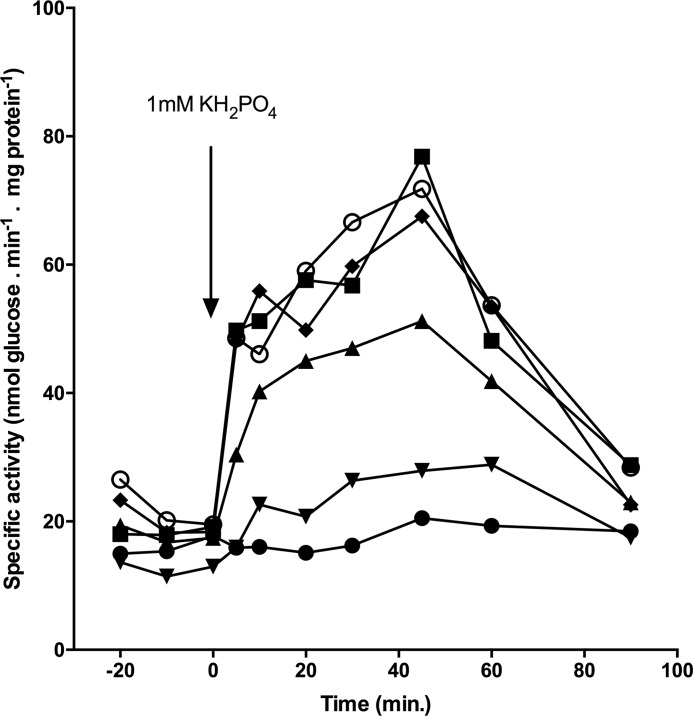
**Trehalase activation after addition of 1 mm phosphate to phosphate-starved cells.** *pho84*Δ (*closed circles*), wild-type (*closed squares*), Tyr^179^-Ala (*closed triangles*), Tyr^179^-Gly (*inverted triangles*), Tyr^179^-Ser (*closed diamond shape*), and Tyr^179^-Phe (*open circles*) are shown.

In addition to its well known transport function, the Pho84 transceptor has the unique property of PKA signaling (17, 18). Previous point mutation studies have shown that these functions can be uncoupled resulting in a non-transporting protein that is still able to activate PKA upon phosphate detection (8). These studies focused on mutating residues that bind P_i_ or that could bind phosphate analogues that trigger PKA activation. In this study, we addressed whether limited release of P_i_ from the Pho84 binding site influenced the ability of Pho84 to activate PKA (Fig. 9). Because transport activity data clearly showed that Tyr^179^ is crucial for Pho84 functionality, trehalase activity assays were performed with relevant mutants.

The ability of the Tyr^179^-Ala mutant strain to activate PKA was slightly reduced, whereas the ability of the Tyr^179^-Gly mutant strain to activate PKA was abolished. The Tyr^179^-Phe and Tyr^179^-Ser mutant strains were able to activate PKA equivalent to the wild-type strain. Modest to strong reductions in transport activity in Asp^178^ and Asp^358^ mutants, respectively, were shown to not affect signaling. Abolishing transport by introducing an Asp^358^-Glu mutation resulted in a 50% reduction in signaling capacity (8). These observations show that, although transport and signaling are tightly intertwined, there are subtle yet profound differences in the residues that regulate the dual functions of Pho84 (Table 2).

**TABLE 2:** **Residues involved in substrate transport and sensing/signaling** The importance of residues in Pho84 for transport and signaling/sensing are as follows: +++, crucial; ++, contributing; +, little contribution. This is based on data obtained by site-directed-mutagenesis and relevant biochemical assays performed in this study and in previous work (8).

Amino acid	Transport	Sensing/signaling
Asp^178^	+	+
Asp^358^	+++	+
Lys^492^	++	+
Tyr^179^	+++	+++

In this study, we showed that Tyr^179^, which is associated with the substrate release step during transport, has a stronger influence on signaling than Asp^358^ (Table 2), which has been shown to be crucial for substrate binding (8). We hypothesize that this difference originates from the different phosphate binding and release mechanisms operating in transport and in signaling.

## Experimental Procedures

### 

#### 

##### Materials and Strains

[^32^P]Orthophosphate (carrier-free) was obtained from PerkinElmer Life Sciences. Anti-Myc-HRP antibodies were obtained from Life Technologies Invitrogen (The Netherlands). Anti-actin-HRP antibodies were obtained from Abcam (UK). All other reagent grade materials were obtained from commercial sources. Haploid, prototrophic *S. cerevisiae* CEN.PK 113–7D (MATa MAL2–8c SUC2) was kindly provided by P. Kötter (Frankfurt, Germany).

##### Sequence Conservation Analysis

Functionally important amino acid residues in the Pho84 primary amino acid sequence were identified by performing a conservation analysis based on MSA using multiple sequence comparison by log-expectation (MUSCLE) alignment software (19). Pho84 homologues were obtained from a BLAST search of the UniProt database. Sequences representative of fungi and plants were selected. The Swiss-Prot protein sequences were retrieved from the NCBI Protein server (www.ncbi.nlm.nih.gov/protein/). The selected species and their accession numbers are *S. cerevisiae* Pho84 (P25297), *P. indica* (A8N031), *Arabidopsis thaliana* Pht1-1 (Q8VYM2), *A. thaliana* Pht 1–2 (Q96243), *Oryza sativa* (Q8GSD9), *Hordeum vulgare* (Q8H6E0), *Medicago truncatula* (Q8GSG4), *Glomus intraradices* (Q96VN6), *Glomus versiforme* (Q00908), and *Pholiota nameko* (Q96X52) (Fig. 2). The MSA figure was created using CLC Workbench (CLC Bio-Qiagen, Aarhus, Denmark). The Tyr^179^ residue was mapped onto the three-dimensional *in silico* Pho84 model (available at PMDB website, ID PM0076296). All of the figures were created using either PyMOL (Molecular Graphics System, version 1.3, Schrödinger, LLC.) or Visual Molecular Dynamics (VMD, version 1.9.1, University of Illinois at Urbana-Champaign, IL) (20).

##### Strain Construction

Mutants were created by oligonucleotide-directed site-specific mutagenesis using a plasmid containing the *PHO84*^WT^ gene as described previously (8). The synthetic oligonucleotides used are listed in Table 3. The mutagenesis was performed using the Stratagene (USA) QuikChange^TM^ II mutagenesis kit according to the manufacturer's protocol. All of the mutant constructs were confirmed by DNA sequencing the entire *PHO84*^WT^ gene. Using pU6H2MYC/*PHO84*^WT^ and pU6H2MYC/*PHO84*^MUT^ as templates for PCR, cassettes containing the last 1.3 kb of *PHO84*^WT^ or *PHO84*^MUT^, c-myc, a His_6_ epitope, and a selection (Kan^r^) marker were transformed into CEN.PK 113–7D and were incorporated into the genome by homologous recombination. Positive transformants were selected on YPD-G418 (200 μg ml^−1^) plates. Resistant colonies were re-streaked onto fresh YPD-G418 (200 μg ml^−1^) plates and were verified by PCR, sequencing, and immunoblot analysis.

**TABLE 3:** **Synthetic oligonucleotides used in site-directed mutagenesis of *PHO84*^WT^**

Amino acid substitution	Mutagenized oligonucleotide (5′ → 3′)
Tyr^179^-Ala	ggtattggtatcggtggtgac**gcc**ccactatcttctattattac
Tyr^179^-Gly	ggtattggtatcggtggtgac**ggc**ccactatcttctattattac
Tyr^179^-Ser	gtattggtatcggtggtgac**tcc**ccactatcttcta
Tyr^179^-Phe	tagaagatagtg**gga**agtcaccaccgataccaatac

##### Growth Conditions

Cells expressing Pho84^WT^-myc or Pho84^MUT^-myc were precultivated aerobically for 12 h in YPD medium (transformed cells were kept in the presence of 200 μg ml^−1^ G418) at 30 °C under continuous agitation, washed twice, and inoculated in synthetic complete (SC) high phosphate (HP_i_, 10 mm KH_2_PO_4_) or low phosphate (LP_i_, 200 μm KH_2_PO_4_) media supplemented with 2% glucose. Cells in LP_i_ conditions were grown aerobically at 30 °C with shaking at 200 rpm for 6 h. Samples were withdrawn at the indicated time points for further phosphate assay and Western blotting analyses.

##### Yeast Growth Spot Tests

Strains were cultured overnight in YPD at 30 °C under continuous agitation. Cells were collected, washed twice with sterile dH_2_O, and resuspended in sterile dH_2_O at an *A*_600_ of 0.3. A ×10 dilution series was spotted onto HP_i_ and LP_i_ SC medium agar plates. The plates were incubated at 30 °C, and growth was recorded every 24 h for a total of 3 days. To visualize the reduction in growth of the *pho84*Δ and mutant strains under LP_i_ conditions, images were acquired with a higher illumination setting resulting in a brighter background. The figures were cropped and no further image processing was applied.

##### Phosphate Transport Measurements

Phosphate uptake in intact *S. cerevisiae* cells expressing Pho84^WT^-myc or Pho84^MUT^-myc was measured as previously described (8). Briefly, cells were grown in LP_i_ medium and assayed by addition of 2 μl of [^32^P]orthophosphate (carrier-free, 0.18 Ci μmol^−1^; 1 mCi = 37 MBq) (PerkinElmer, USA) and phosphate (10 mm KH_2_PO_4_) to a final concentration of 220 μm. To determine total transport activity, a final phosphate concentration of 110 μm was used. Cells were resuspended to 1 mg ml^−1^ (wet weight) in buffer containing 25 mm Tris succinate, pH 4.5, and 3% glucose. Aliquots of 30 μl were incubated for 10 min at room temperature. After 10 min, 3 ml of ice-cold 25 mm Tris succinate, pH 4.5, buffer was added to stop the reaction. The cells were rapidly filtered (Whatman GF/F, USA) and the radioactivity retained on the filters was measured by liquid scintillation spectrometry.

##### Immunoblot Analysis of Pho84 Expression

Immunoblot analysis was performed as previously described (8). Briefly, membrane fractions were collected and 10 μg of protein samples were separated by SDS-PAGE using a 10% Laemmli system (21). Anti-myc HRP-conjugated antibody (anti-myc-HRP, 1:5000, Novex®) was used to detect expression of the Pho84^WT/MUT^-myc constructs. The (−) control was cut from the blot to avoid visualization of irrelevant samples, but detection was performed simultaneously with the mutant sample blot. As a loading control, 10 μg of total cell extract was loaded onto a separate gel according to the previously described protocol, and anti-β-actin HRP-conjugated antibody (1:5000) (Abcam, UK) was used for detection. After 1 min of incubation with chemiluminescent substrate (GE Healthcare, UK), the membrane-enriched sample blot was exposed to X-ray film for 1.5 min. The control blot was visualized using the Bio-Rad ChemiDoc^TM^ MP imaging system with an exposure time of 30 s. The molecular masses of the separated proteins were determined by their mobility relative to the pre-stained protein markers (Fermentas, Germany). Figures were cropped and no further image processing was applied.

##### Acid Phosphatase Assays

rAPase activity was assayed in liquid using an adapted protocol for the colorimetric Abcam® Acid Phosphatase Assay Kit. Briefly, whole cells were used as the source of the enzyme and *p*-nitrophenyl phosphate was used as the substrate. Yeast strains were grown overnight in 5 ml of YPD at 30 °C, centrifuged, and washed twice with LP_i_ SC medium. Washed cells were inoculated into 15 ml of LP_i_ or HP_i_ phosphate SC medium to an *A*_600_ of 0.4. Cells were grown at 30 °C under continuous agitation for 3 h after which 80 μl of the cell suspension was harvested, washed once with acetate buffer (60 mm, pH 4.5), and resuspended in 80 μl of acetate buffer containing 1 mm *p*-nitrophenyl phosphate (final concentration). The reaction was incubated at 25 °C for 1 h and was then stopped with the addition of 20 μl of saturated Na_2_CO_3_. The cells were removed from the reaction by centrifugation before measuring the *A*_405_. The relative rAPase activity was determined by the formula *A*_405_/*A*_600_ × *t*, in which *t* is the time of incubation (min).

##### Trehalase Activity Measurements

Cells were cultured at 30 °C to exponential phase (*A*_600_ = 1.0–1.5) in YP medium with 2% (w/v) glucose. Mid-exponential phase cells were harvested and transferred to phosphate starvation medium (5.7 g liter^−1^ YNB without phosphate, with ammonium sulfate) with 4% (w/v) glucose and appropriate auxotrophic supplements. Cells were starved of phosphate for 3 days at 30 °C under continuous shaking, and starvation medium was refreshed daily. The phosphate-starved glucose-repressed cells were rapidly cooled on ice and harvested by centrifugation (5000 × *g* for 5 min at 4 °C). The pellet was washed twice with ice-cold 25 mm MES buffer, pH 6.0, resuspended in phosphate starvation medium with 4% (w/v) glucose, and incubated at 30 °C with shaking. After 30 min of incubation, 1 mm KH_2_PO_4_ was added to the culture. 75 mg ml^−1^ cell samples were taken at the indicated time points. Cells were rapidly cooled by the addition of ice-cold dH_2_O, centrifuged (5000 × *g* for 5 min at 4 °C), and re-suspended in 0.5 ml of ice-cold 25 mm MES buffer, pH 7.0. Crude cell extracts were prepared as described previously (22) and dialyzed (BRL microdialysis system) against 25 mm MES buffer, pH 7.0, with 50 μm CaCl_2_ at 4 °C. Trehalase activity in the dialyzed cell extracts was determined using a coupled enzymatic reaction of glucose oxidase and peroxidase with glucose as described previously (22). The specific activity was expressed as nanomole of glucose liberated per min per mg of protein. The total amount of protein in the samples was determined using the standard Lowry method described previously (23).

##### Computational Section

An inward-facing open conformation of Pho84 from *S. cerevisiae* was obtained by structurally fitting (7) it to the helix containing the transmembrane region (TM-I–XII, Table 1) in the crystal structure of the glycerol 3-phosphate/phosphate antiporter GlpT (24) from *E. coli*. This conformation was used as the starting conformation for the series of MD simulations performed in this work. After performing primary structural alignments between Pho84 (587 amino acids) and GlpT, the N- and C-terminal extensions of Pho84 could not be modeled onto the GlpT structure that contains only 452 amino acids. Hence, we decided not to include the regions of Pho84 (amino acids 1–54 and 552–587) that we could not structurally model in these studies. It must, however, be noted that these regions of Pho84 may be important for biological function and will be the target of future studies.

To investigate the role of proton transfer on translocation of P_i_ by Pho84, simulations were performed after protonating Asp^178^, which was previously suggested (8) to participate in the intrinsic proton transfer system of Pho84. A model of the truncated Pho84 embedded in a phospholipid bilayer was constructed after inserting Pho84 into a fully solvated (0.15 m TIP3P/water solution of potassium chloride) 1-palmitoyl-2-oleoyl-*sn*-glycero-3-phosphocholine bilayer using the CHARMM-gui (25).

Systems were also set up where a single inorganic phosphate ligand (H_3_PO_4_, H_2_PO_4_^−^, and HPO_4_^2−^) was inserted into the center of Pho84 between two amino acids believed to be a part of the binding pocket (Lys^492^) and the proton transfer system (Asp^178^) using PACKMOL (26). This positioning was based on a recent X-ray crystal structure in which PiPT was co-crystallized with P_i_ (5). During the system design, titratable amino acids were assigned charges based upon neutral pH conditions. All of the MD simulations were performed using Amber software (version 10, USCF, San Francisco, CA) (27, 28). The Amber14SB force field, which is an evolved continuation of the Amber99SB (29) force field with improved protein backbone parameters, was used with compatible force fields, such as the newly developed lipid force field Lipid14 (30) and GAFF, a force field that has been developed for small organic ligands (31). The parameters used for the simulations of explicitly solvated potassium and chloride ions were those developed by Joung and Cheatham (32). The starting geometries for H_3_PO_4_, H_2_PO_4_^−^, and HPO_4_^2−^ were initially built in Avogadro (33), were pre-minimized using MMFF94 (34), and were further optimized in Gaussian09 (35) using the default convergence criteria implemented in the software at the HF/6–31G* level. GAFF parameters were used for simulations that incorporated H_3_PO_4_, whereas recently updated parameters for bioorganic phosphates were used for simulations that incorporated H_2_PO_4_^−^ and HPO_4_^2−^ (36). The atomic partial charges for the P_i_ ligands were estimated using the restrained electrostatic potential procedure (37) and the Antechamber module.

##### Unrestrained Molecular Dynamics Simulations

Each system was initially energetically minimized by removing the high-energy van der Waals contacts with a 500.0 kcal mol^−1^ Å^−2^ position restraint on the lipids, Pho84, and the P_i_ ligand. Subsequently, the restraint for the lipids was removed. In a final energy minimization step, the whole system was allowed to relax without restraints. In this study, a total number of 10,000 steps were undertaken in each round and were divided into 5,000 steps of steepest descent and 5,000 steps of conjugate gradient. In a second step, the temperature of the system was initially raised from 0 to 100 K for 5 ps under conditions of NVT (constant number of particles, volume, and temperature) imposing a 10.0 kcal mol^−1^ Å^−2^ restraint on the lipids, Pho84, and the P_i_ ligand before running an additional 100 ps of simulation under conditions of NPT (constant number of particles, pressure, and temperature) in which the temperature was further raised from 100 to 310 K. The NPT simulation step was conducted using an anisotropic pressure scaling and a pressure relaxation constant (τ_p_) of 2.0 ps while still applying a 10.0 kcal mol^−1^ Å^−2^ restraint on the lipid molecules, Pho84, and the P_i_ ligand. Subsequently, the restraint on the lipids was released and an additional 30 ns of simulation data using a τ_p_ of 1.0 ps was collected. During all of the simulations, the temperature was held constant using Langevin dynamics with a collision frequency set to 1.0 ps^−1^. All of the bonds to hydrogen were constrained using the SHAKE algorithm, which allowed a time step set to 0.002 ps. Periodic boundary conditions were applied in all of the directions using a 10-Å non-bonded interaction cutoff. Long-range electrostatics were treated using the particle mesh Ewald summation method, and long-range van der Waals interactions were corrected using a continuum model correction of both energy and pressure. Data were collected for the membrane system in the absence and presence of the P_i_ ligand, as well as for systems studying the effect of Asp^178^ protonation/deprotonation. Data were generally saved every 10 ps.

##### Steered Molecular Dynamics Simulations

To study the cytosolic release of P_i_ from the inward-facing open conformation of Pho84, constant velocity steered MD (14) was used to obtain potential release pathways and their respective work profiles. Initially, equilibration at NPT (1 bar, 310 K) and a restraint of 500.0 kcal mol^−1^ Å^−2^ was imposed on the distance between the phosphorus atom of P_i_ and the ϵ-amino nitrogen of Lys^492^, which set the distance 0.2 Å shorter than the equilibrated distance found after 30 ns of unrestrained simulation. To investigate how the residual motion of the restrained Lys^492^-P_i_ pair and the overall dynamics of Pho84 affect the various cytosolic release pathways, five individual simulations were conducted for each system after initially restraining the Lys^492^-P_i_ distance for 0.1, 0.2, 0.5, 1.0, or 5.0 ns as described previously. Numerous constant velocity steered MD simulations were then performed using a moving harmonic potential with a spring constant of 5.68 kcal mol^−1^ Å^−2^ (790 pN Å^−1^) and increasing the distance between Lys^492^-P_i_ at a constant pulling velocity of 10 Å ns^−1^. The total extended distance studied in each simulation was 35 Å in which the atomic coordinates and forces were saved every 1 ps. Although, in principle, a steered MD simulation can be considered analogous to a single-molecule atomic force microscopy experiment, the pulling velocity commonly employed is typically several orders of magnitude faster, thus it is difficult to make direct comparisons between calculated ligand pulling work profiles using these techniques (38). Typically, constant velocity steered MD simulations have utilized different pulling velocities to optimize the conditions under which the ligand experiences amino acid contacts on the way out of the receptor site and to ensure an energetically stable trajectory. The constant velocity chosen in this study (0.01 Å ns^−1^) is among the slowest found in the literature and was selected based on the results of unbinding studies on similar systems (39–42).

## Author Contributions

D. S. and J. V. d. V. performed the site-directed mutagenesis, phosphate uptake assays, acid phosphatase activity studies, and the spot tests. G. V. Z. performed the trehalase activity measurements. B. C. G. K. performed all of the theoretical calculations and analyses. B. C. G. K., D. S., and B. P. conceived the idea and the experimental design of the project. B. C. G. K. and D. S. led the writing of this paper. All of the authors have taken part in the preparation of this manuscript, have reviewed the results, and have approved the final version of this manuscript.

## Supplementary Material

Supplemental Data

## Footnotes

4 The abbreviations used are:

MFS: major facilitator superfamily
MD: molecular dynamics
SMD: steered molecular dynamics
MSA: multiple sequence alignment
rAPase: repressible acid phosphatase
HP_i_: high-P_i_
LP_i_: low-P_i_.
