# Supplementary material for: Key Residues and Phosphate Release Routes in the Saccharomyces cerevisiae Pho84 Transceptor: THE ROLE OF TYR179 IN FUNCTIONAL REGULATION
Source: J Biol Chem. 2016 Nov 8;291(51):26388–98. doi: 10.1074/jbc.M116.738112 (PMC5159500; doi:10.1074/jbc.M116.738112)
Supplement: Supplemental Data [file supp_291_51_26388__index.html]

Key Residues and Phosphate Release Routes in the Saccharomyces cerevisiae Pho84 Transceptor - The Role of Tyr179 in Functional Regulation — Key Residues and Phosphate Release Routes in the Saccharomyces cerevisiae Pho84 Transceptor — Mechanistic Studies on the Pho84 Transporter — Supplemental Data 

# Key Residues and Phosphate Release Routes in the *Saccharomyces cerevisiae* Pho84 Transceptor

## Supplemental Data

- Supporting\_Information (.pdf, 33.4 MB) - Supporting Information
