# Supplementary material for: Key Residues and Phosphate Release Routes in the Saccharomyces cerevisiae Pho84 Transceptor: THE ROLE OF TYR179 IN FUNCTIONAL REGULATION
Source: J Biol Chem. 2016 Nov 8;291(51):26388–98. doi: 10.1074/jbc.M116.738112 (PMC5159500; doi:10.1074/jbc.M116.738112)
Supplement: Supplemental Data [file 10.1074_M116.738112_jbc.M116.738112-1.pdf]

Supporting Information for:

Key Residues and Phosphate Release Routes in the *Saccharomyces cerevisiae* Pho84 Transceptor – The Role of Tyr^{179} in Functional Regulation

Dieter R. Samyn,[2,†] Jeroen van der Veken,[2,§] Griet Van Zeebroeck,[3,4] Bengt L. Persson,[2] Björn C. G. Karlsson[1,2,]*

[1] Computational Chemistry & Biochemistry Group. [2] Linnæus University Centre for Biomaterials Chemistry, Linnæus University, SE-391 82 Kalmar, Sweden. [3] Department of Molecular Microbiology, VIB, Kasteelpark Arenberg 31, BE-3001 Leuven-Heverlee, Flanders, Belgium. [4] Laboratory of Molecular Cell Biology, Institute of Botany and Microbiology, Katholieke Universiteit Leuven, Kasteelpark Arenberg 31, BE-3001 Leuven-Heverlee, Flanders, Belgium.

Present addresses:
† Concordia University, Loyola Campus, Biology Department, 7141 Sherbrooke St W, Montréal, H4B 1R6, QC Canada.

§ Plant Sciences Unit, Applied Genetics and Breeding, Institute for Agricultural and Fisheries Research (ILVO), Caritasstraat 39, BE-9090 Melle, Belgium.

* Correspondence to:
Björn C.G. Karlsson
E-mail: bjorn.karlsson@lnu.se
Tel: +46-480-446740
Fax: +46-480-446262

Table of Contents

Table of Contents	Name	Page(s)
**Molecular Dynamics (MD) System Design**		
Box Dimensions and Total Number of Molecules/Residues:	*Table S1*	*S3*
**Unrestrained MD Simulation Data**		
Potential, Kinetic & Total Energy:		
Pho84	*Figure S1*	*S4*
Pho84 – Asp178-H	*Figure S2*	*S5*
Pho84 – neu	*Figure S3*	*S6*
Pho84 – neu – Asp178-H	*Figure S4*	*S7*
Pho84 – minus	*Figure S5*	*S8*
Pho84 – minus – Asp178-H	*Figure S6*	*S9*
Pho84 – 2minus	*Figure S7*	*S10*
Pho84 – 2minus – Asp178-H	*Figure S8*	*S11*
Temperature & Density:		
Pho84	*Figure S9*	*S12*
Pho84 – Asp178-H	*Figure S10*	*S13*
Pho84 – neu	*Figure S11*	*S14*
Pho84 – neu – Asp178-H	*Figure S12*	*S15*
Pho84 – minus	*Figure S13*	*S16*
Pho84 – minus – Asp178-H	*Figure S14*	*S17*
Pho84 – 2minus	*Figure S15*	*S18*
Pho84 – 2minus – Asp178-H	*Figure S16*	*S19*

Transmembrane Helix RMSD — *Figure S17* — *S20*

Atomic Distances
P_i-Lys^{492} & P_i-Asp^{178}/P_i-Asp^{178}-H — *Figure S18* — *S21*
Asp^{178}/Asp^{178}-H-Asp^{76} — *Figure S19* — *S22*
P_i-Tyr^{179} — *Figure S20* — *S23*

Steered Molecular Dynamics (SMD) Simulation Data

Force Profiles:
Pho84 – neu / Pho84 – minus – Asp178-H / Pho84 – minus — *Figure S21* — *S24*
Pho84 – 2minus — *Figure S22* — *S25*

Work Profiles:
Pho84 – neu / Pho84 – minus – Asp178-H / Pho84 – minus — *Figure S23* — *S26*
Pho84 – 2minus — *Figure S24* — *S26*

Pho84 Residue-P_i Hydrogen Bonding:
Pho84 – neu — *Table S2* — *S27*
Pho84 – minus – Asp178-H — *Table S3* — *S28*
Pho84 – minus — *Table S4* — *S29*
Pho84 – 2minus — *Table S5* — *S30*

Pho84 Residue-P_i Hydrogen Bond Formation and Disruption Lifetime:
Pho84 – neu — *Figure S25* — *S31-S32*
Pho84 – minus – Asp178-H — *Figure S26* — *S33-S34*
Pho84 – minus — *Figure S27* — *S35-S36*
Pho84 – 2minus — *Figure S28* — *S36*

Water-P_i Hydrogen Bonding:
Pho84 – neu / Pho84 – minus – Asp178-H / Pho84 – minus — *Figure S29* — *S37*
Pho84 – 2minus — *Figure S30* — *S38*

Release Routes:
Pho84 – neu — *Figure S31* — *S39-S40*
Pho84 – minus – Asp178-H — *Figure S32* — *S41-S42*
Pho84 – minus — *Figure S33* — *S43-S44*
Pho84 – 2minus — *Figure S34* — *S44*

MD System Design

Box Dimensions and Total Number of Molecules/Residues:

Table S1. Total number of molecules added in each system and final equilibrated box dimensions

		Number of molecules/residues					
**System**	**Box size$_{t=30ns}$ (x*y*z /Å^3)^a**	**Pho84**	**P_i**	**K^+**	**Cl^-**	**POPC**	**Water**
Pho84 (without P_i in the binding site)	99.65×96.16×113.31	497	-	48	48	247	23316
Pho84 & protonated Asp[178]	97.34×98.33×113.48	497	-	48	49	247	23315
Pho84 & H_3PO_4	96.92×98.64×113.62	497	1	48	48	247	23316
Pho84 & H_3PO_4 & protonated Asp[178]	104.24×90.70×114.82	497	1	48	49	247	23315
Pho84 & $H_2PO_4^-$	95.48×99.48×114.04	497	1	49	48	247	23315
Pho84 & $H_2PO_4^-$ & protonated Asp[178]	99.66×95.77×113.72	497	1	49	49	247	23314
Pho84 & HPO_4^{2-}	98.22×99.11×111.50	497	1	50	48	247	23315
Pho84 & HPO_4^{2-} & protonated Asp[178]	106.17×89.68×113.99	497	1	50	49	247	23314

^{a} Box size$_{t=0}$ = 100.11×100.11×118.27Å^3

Unrestrained MD Simulation Data

Potential, Kinetic, & Total Energy:

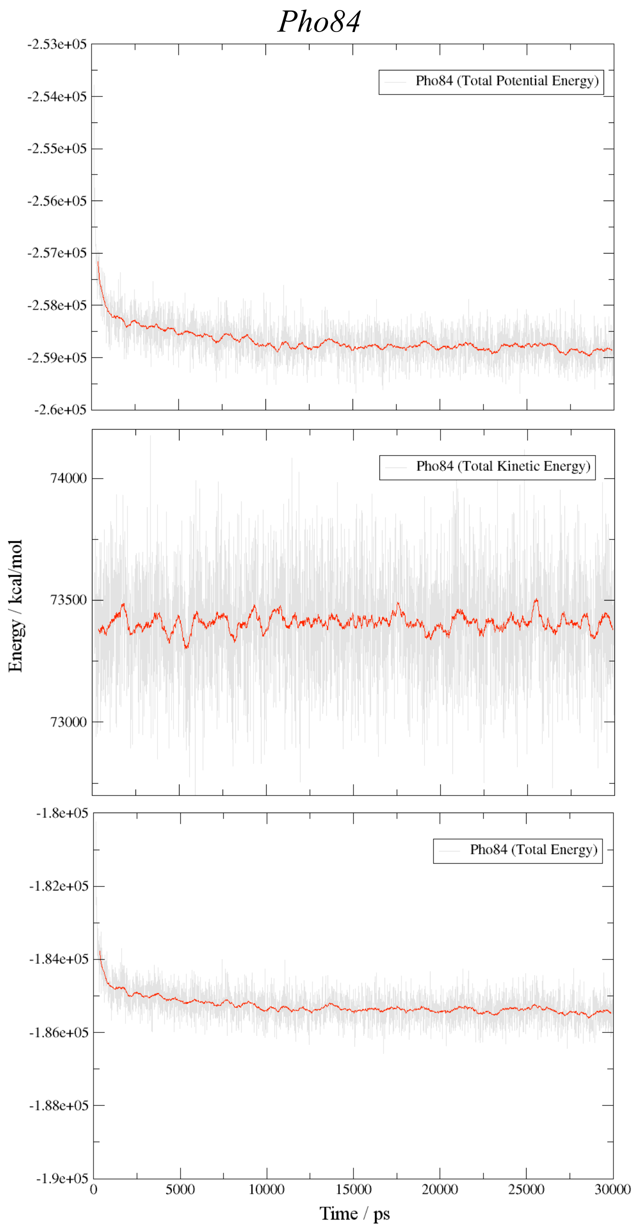

Figure S1. MD equilibration data extracted from a 30 ns simulation of fully solvated 1-palmitoyl-2-oleoyl-*sn*-glycero-3-phosphocholine (POPC) bilayer-embedded Pho84 without P_i in the binding site (Pho84). Potential, kinetic, and total energy (kcal/mol) fluctuations over time (ps; in gray) and a 50 data point running average (in red) are shown.

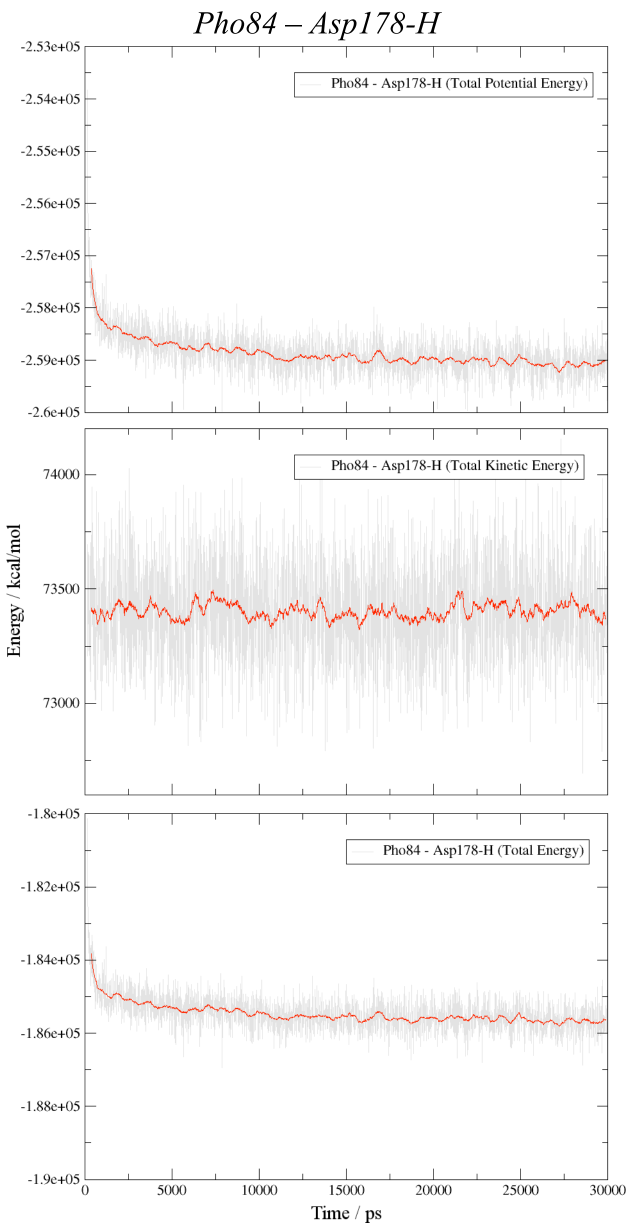

Figure S2. MD equilibration data extracted from a 30 ns simulation of fully solvated 1-palmitoyl-2-oleoyl-*sn*-glycero-3-phosphocholine (POPC) bilayer-embedded Pho84 with a protonated Asp178 and without P_i in the binding site (Pho84 – Asp178-H). Potential, kinetic, and total energy (kcal/mol) fluctuations over time (ps; in gray) and a 50 data point running average (in red) are shown.

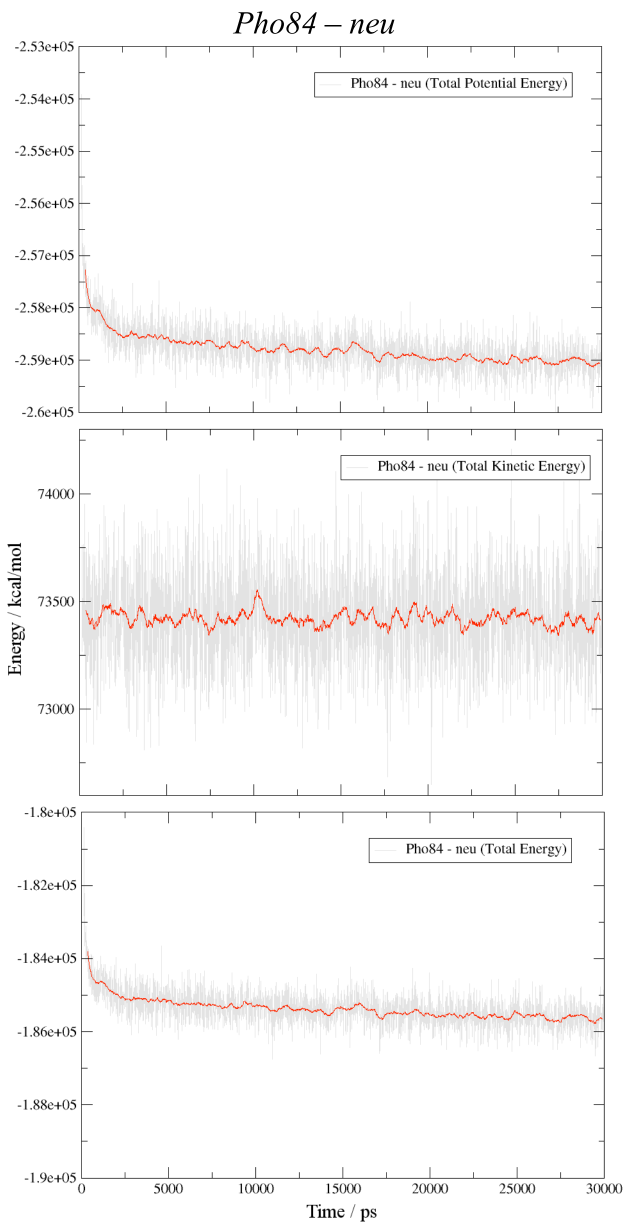

Figure S3. MD equilibration data extracted from a 30 ns simulation of fully solvated 1-palmitoyl-2-oleoyl-*sn*-glycero-3-phosphocholine (POPC) bilayer-embedded Pho84 with neutral H_3PO_4 in the binding site (Pho84 – neu). Potential, kinetic, and total energy (kcal/mol) fluctuations over time (ps; in gray) and a 50 data point running average (in red) are shown.

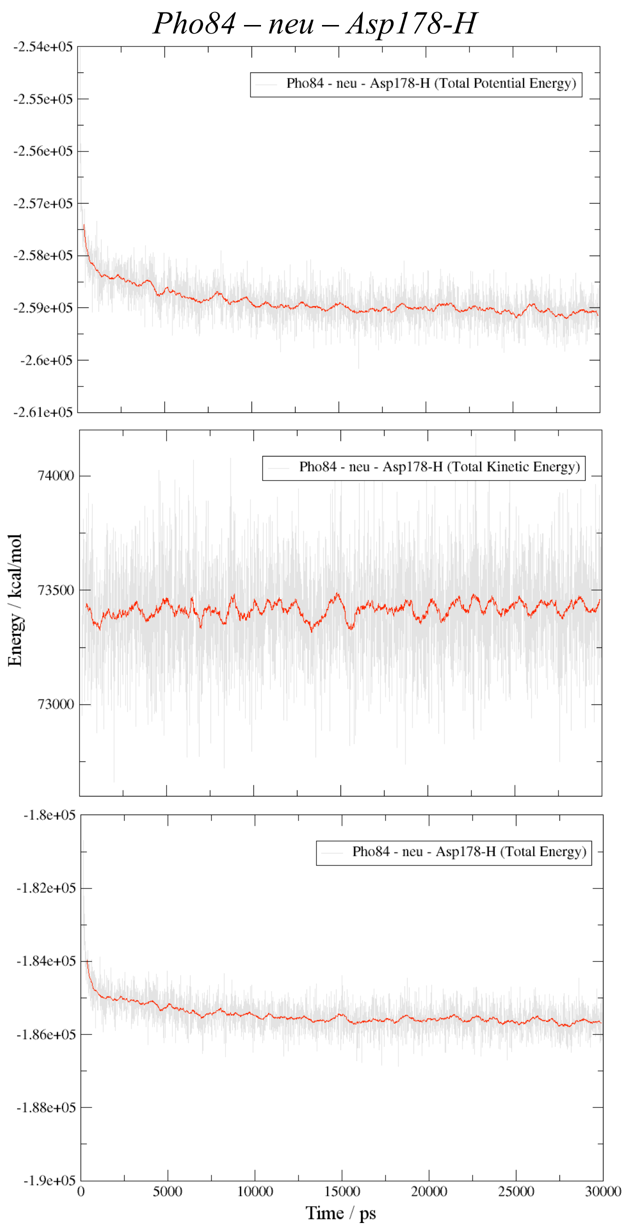

Figure S4. MD equilibration data extracted from a 30 ns simulation of fully solvated 1-palmitoyl-2-oleoyl-*sn*-glycero-3-phosphocholine (POPC) bilayer-embedded Pho84 with protonated Asp178 and with neutral H_3PO_4 in the binding site (Pho84 – neu – Asp178-H). Potential, kinetic, and total energy (kcal/mol) fluctuations over time (ps; in gray) and a 50 data point running average (in red) are shown.

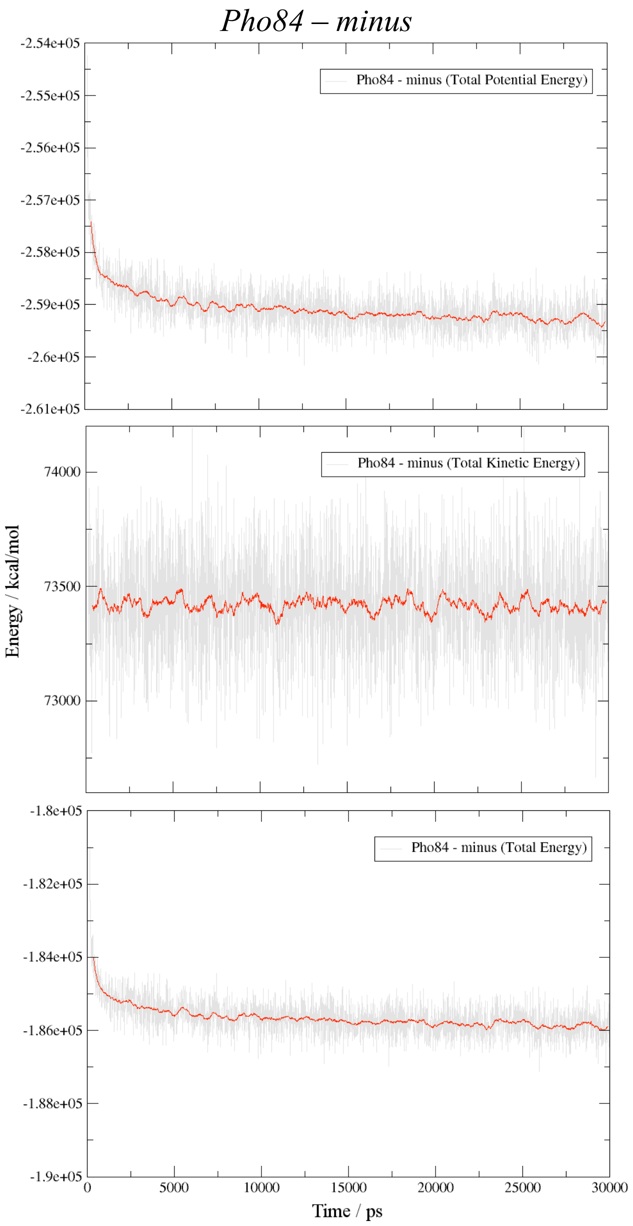

Figure S5. MD equilibration data extracted from a 30 ns simulation of fully solvated 1-palmitoyl-2-oleoyl-*sn*-glycero-3-phosphocholine (POPC) bilayer-embedded Pho84 with the single-charged phosphate $H_2PO_4^-$ in the binding site (Pho84 – minus). Potential, kinetic, and total energy (kcal/mol) fluctuations over time (ps; in gray) and a 50 data point running average (in red) are shown.

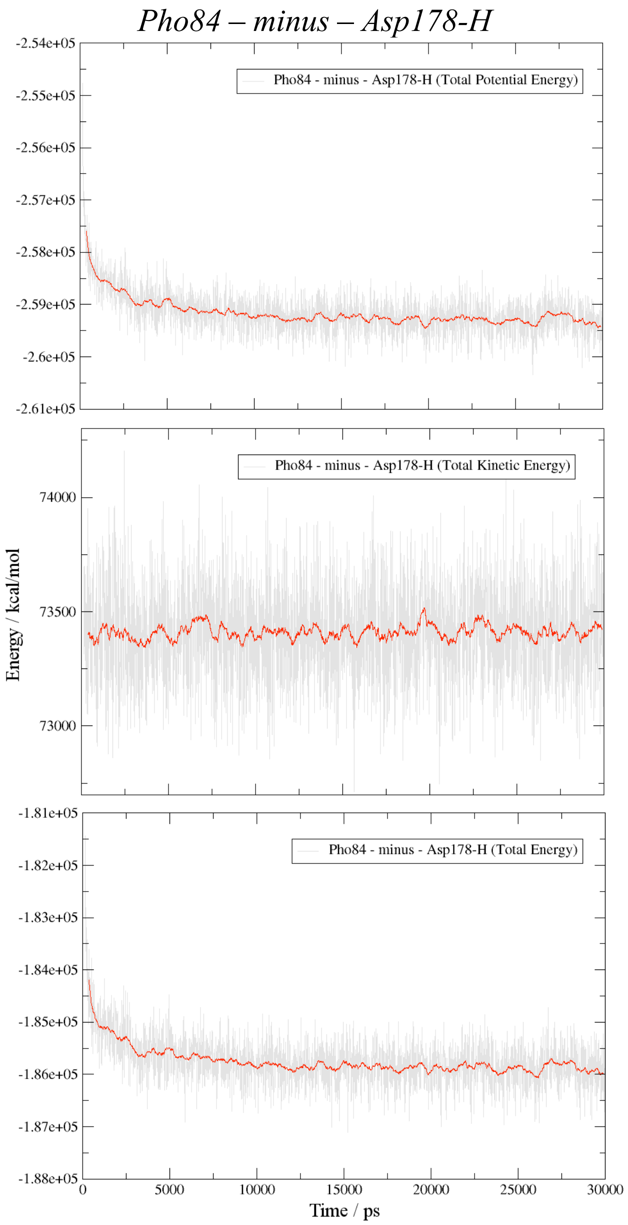

Figure S6. MD equilibration data extracted from a 30 ns simulation of fully solvated 1-palmitoyl-2-oleoyl-*sn*-glycero-3-phosphocholine (POPC) bilayer-embedded Pho84 with protonated Asp178 and with the single-charged phosphate $H_2PO_4^-$ in the binding site (Pho84 – minus – Asp178-H). Potential, kinetic, and total energy (kcal/mol) fluctuations over time (ps; in gray) and a 50 data point running average (in red) are shown.

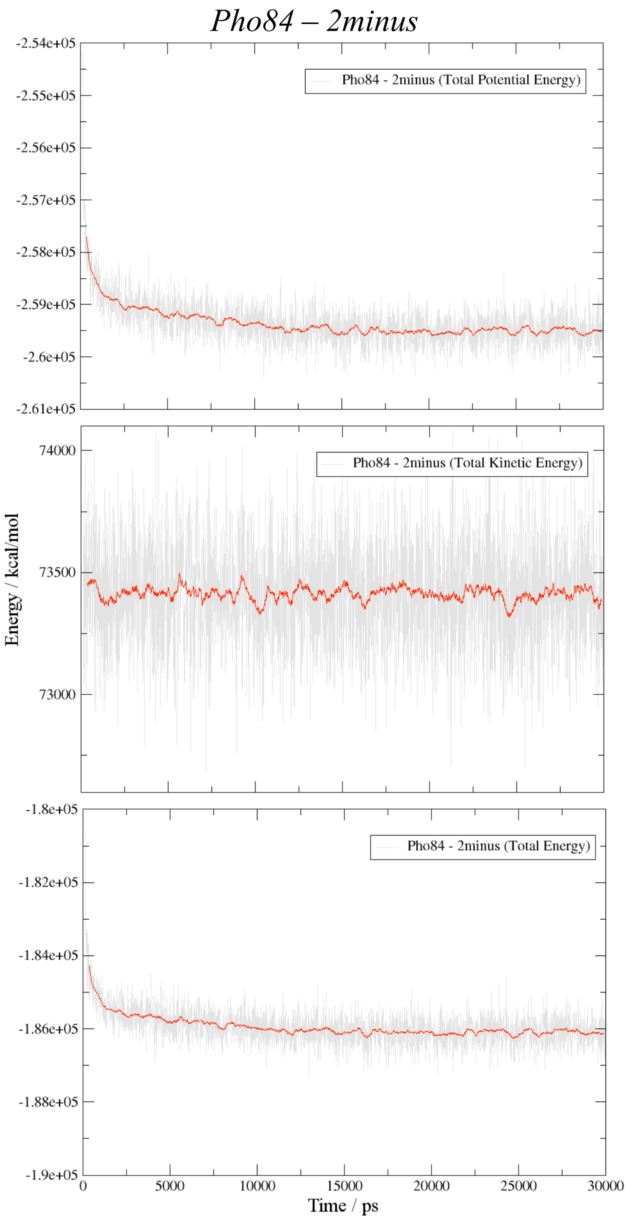

Figure S7. MD equilibration data extracted from a 30 ns simulation of fully solvated 1-palmitoyl-2-oleoyl-*sn*-glycero-3-phosphocholine (POPC) bilayer-embedded Pho84 with the double-charged phosphate HPO_4^{2-} in the binding site (Pho84 – 2minus). Potential, kinetic, and total energy (kcal/mol) fluctuations over time (ps; in gray) and a 50 data point running average (in red) are shown.

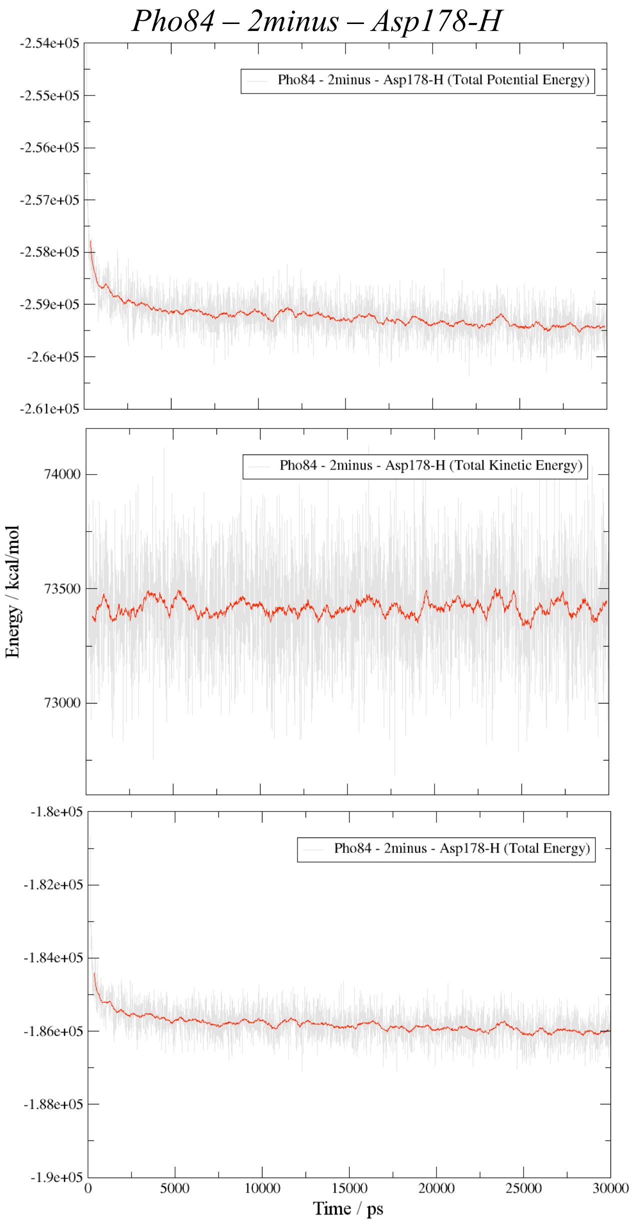

Figure S8. MD equilibration data extracted from a 30 ns simulation of fully solvated 1-palmitoyl-2-oleoyl-*sn*-glycero-3-phosphocholine (POPC) bilayer-embedded Pho84 with protonated Asp178 and with the double-charged phosphate HPO_4^{2-} in the binding site (Pho84 – 2minus – Asp178-H). Potential, kinetic, and total energy (kcal/mol) fluctuations over time (ps; in gray) and a 50 data point running average (in red) are shown.

Temperature & Density

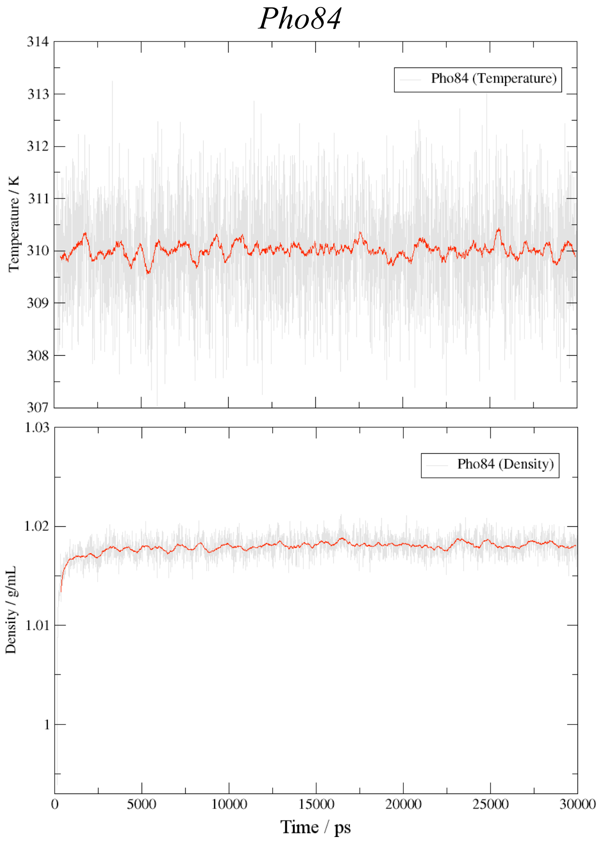

Figure S9. MD equilibration data extracted from a 30 ns simulation of fully solvated 1-palmitoyl-2-oleoyl-*sn*-glycero-3-phosphocholine (POPC) bilayer-embedded Pho84 without P_i in the binding site (Pho84). Fluctuations in temperature (K) and density (g/mL) over time (ps; in gray) and a 50 data point running average (in red) are shown.

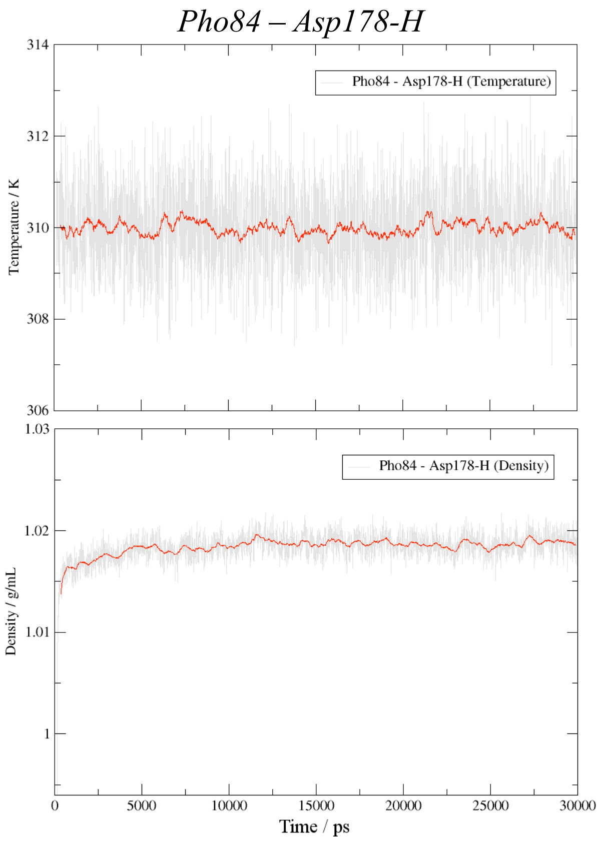

Figure S10. MD equilibration data extracted from a 30 ns simulation of fully solvated 1-palmitoyl-2-oleoyl-*sn*-glycero-3-phosphocholine (POPC) bilayer-embedded Pho84 with protonated Asp178, but without P_i in the binding site (Pho84 – Asp178-H). Fluctuations in temperature (K) and density (g/mL) over time (ps; in gray) and a 50 data point running average (in red) are shown.

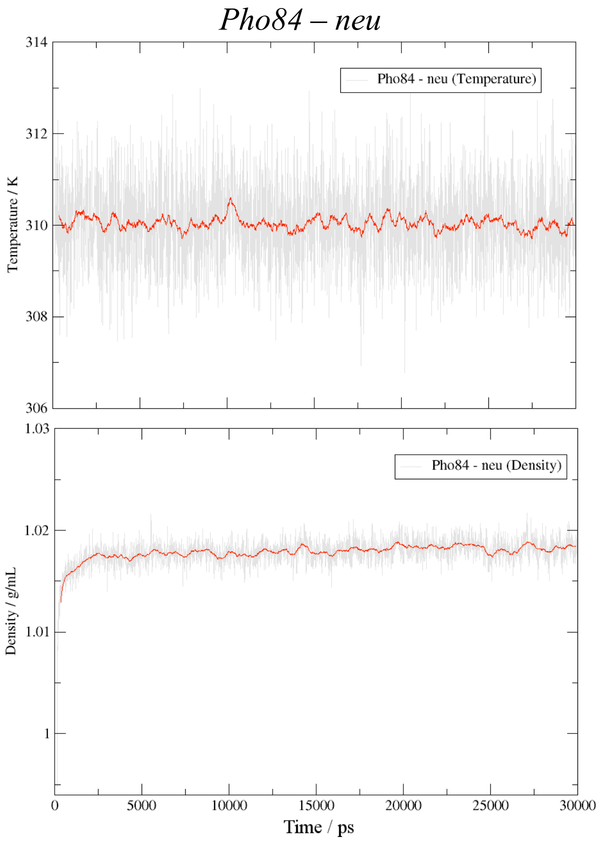

Figure S11. MD equilibration data extracted from a 30 ns simulation of fully solvated 1-palmitoyl-2-oleoyl-*sn*-glycero-3-phosphocholine (POPC) bilayer-embedded Pho84 with neutral H_3PO_4 in the binding site (Pho84 – neu). Fluctuations in temperature (K) and density (g/mL) over time (ps; in gray) and a 50 data point running average (in red) are shown.

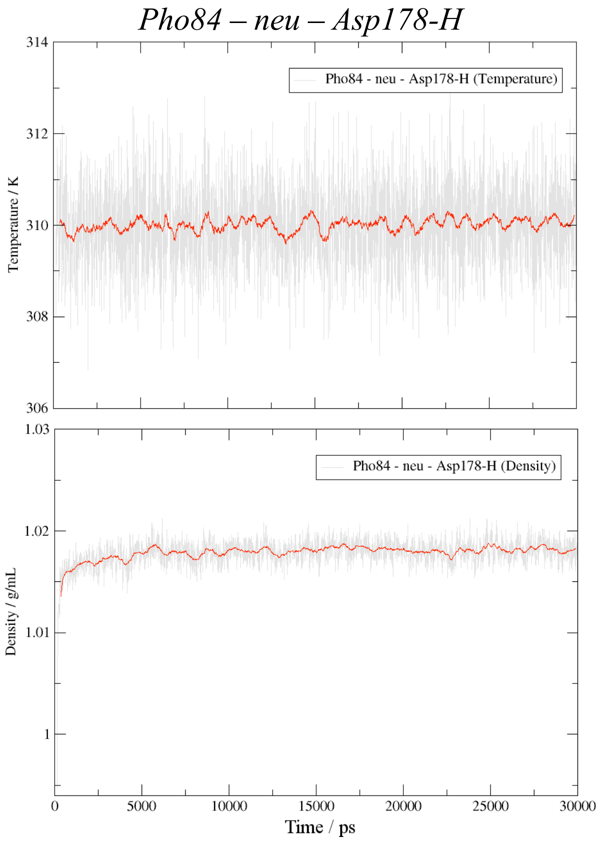

Figure S12. MD equilibration data extracted from a 30 ns simulation of fully solvated 1-palmitoyl-2-oleoyl-*sn*-glycero-3-phosphocholine (POPC) bilayer-embedded Pho84 with protonated Asp178 and with neutral H_3PO_4 in the binding site (Pho84 – neu – Asp178-H). Fluctuations in temperature (K) and density (g/mL) over time (ps; in gray) and a 50 data point running average (in red) are shown.

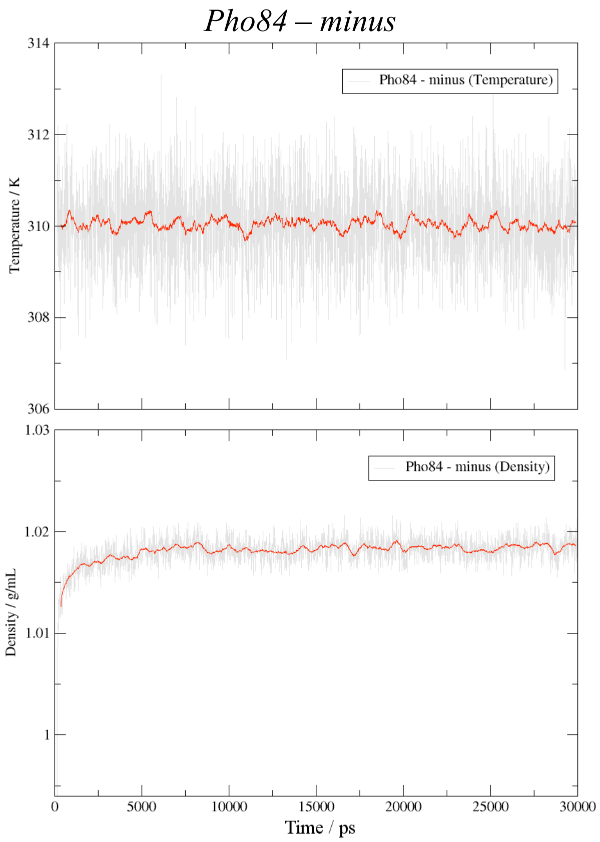

Figure S13. MD equilibration data extracted from a 30 ns simulation of fully solvated 1-palmitoyl-2-oleoyl-*sn*-glycero-3-phosphocholine (POPC) bilayer-embedded Pho84 with single-charged phosphate $H_2PO_4^-$ in the binding site (Pho84 – minus). Fluctuations in temperature (K) and density (g/mL) over time (ps; in gray) and a 50 data point running average (in red) are shown.

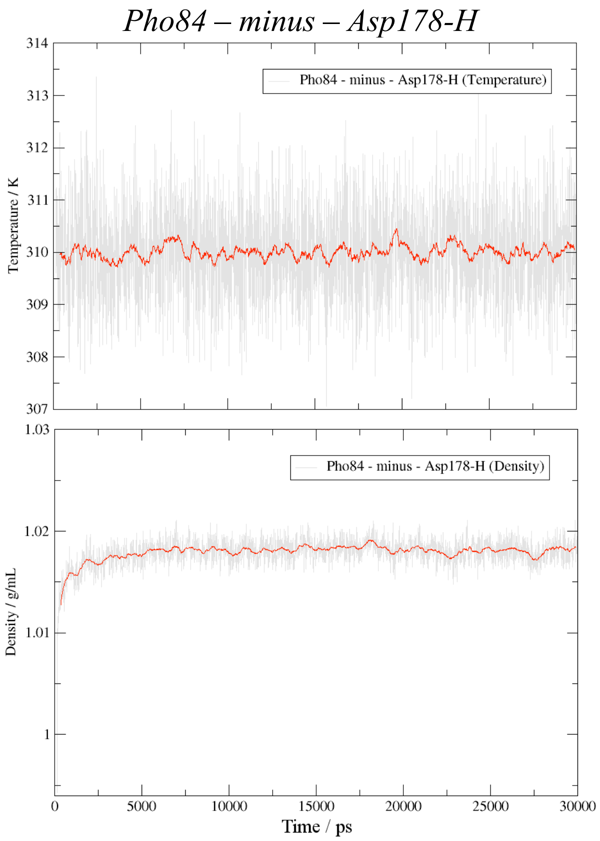

Figure S14. MD equilibration data extracted from a 30 ns simulation of fully solvated 1-palmitoyl-2-oleoyl-*sn*-glycero-3-phosphocholine (POPC) bilayer-embedded Pho84 with protonated Asp178 and with single-charged phosphate $H_2PO_4^-$ in the binding site (Pho84 – minus – Asp178-H). Fluctuations in temperature (K) and density (g/mL) over time (ps; in gray) and a 50 data point running average (in red) are shown.

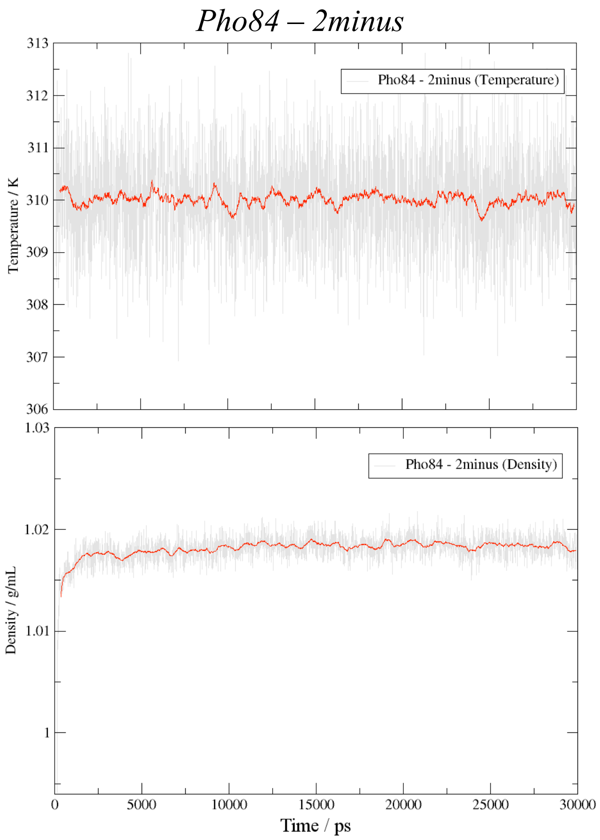

Figure S15. MD equilibration data extracted from a 30 ns simulation of fully solvated 1-palmitoyl-2-oleoyl-*sn*-glycero-3-phosphocholine (POPC) bilayer-embedded Pho84 with double-charged phosphate HPO_4^{2-} in the binding site (Pho84 – 2minus). Fluctuations in temperature (K) and density (g/mL) over time (ps; in gray) and a 50 data point running average (in red) are shown.

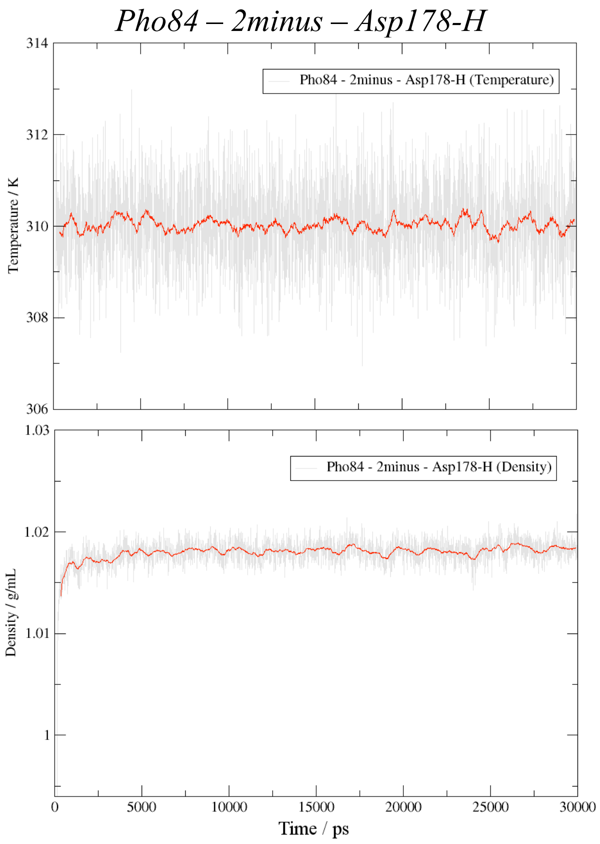

Figure S16. MD equilibration data extracted from a 30 ns simulation of fully solvated 1-palmitoyl-2-oleoyl-*sn*-glycero-3-phosphocholine (POPC) bilayer-embedded Pho84 with protonated Asp178 and with double-charged phosphate HPO_4^{2-} in the binding site (Pho84 – 2minus – Asp178-H). Fluctuations in temperature (K) and density (g/mL) over time (ps; in gray) and a 50 data point running average (in red) are shown.

Transmembrane Helix RMSD

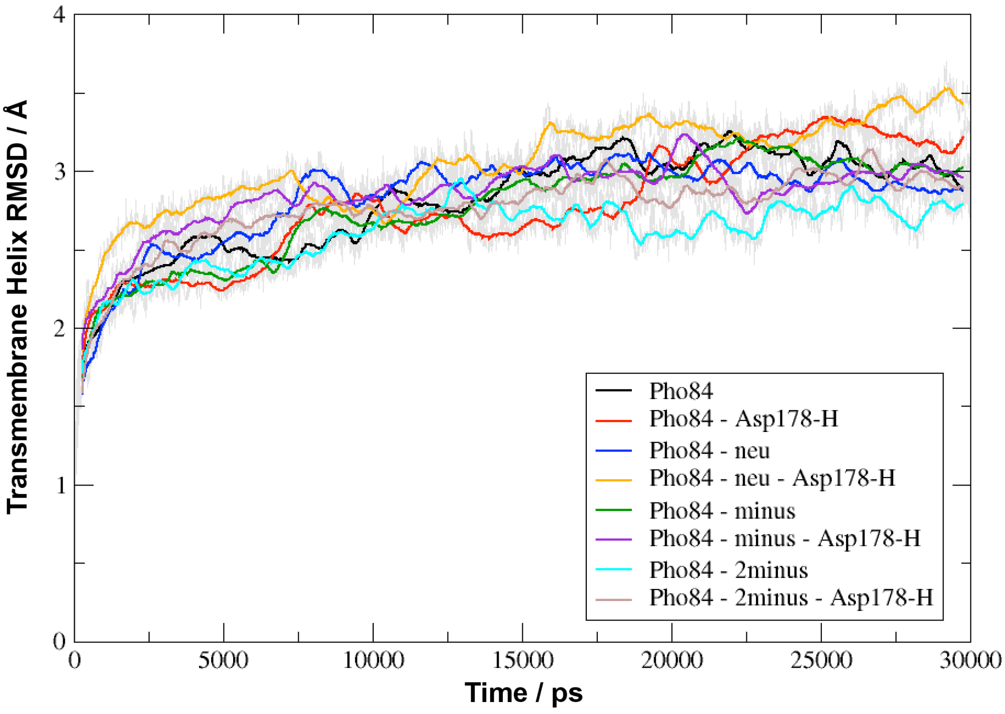

Figure S17. The RMSDs of the backbone (N, Cα, and C atoms) of the transmembrane helices in various Pho84 simulations using 30 ns of equilibration (in gray) and a 50 point running average. Residues belonging to transmembrane regions are presented in Table 1 in the main paper and were previously proposed by Lagerstedt and co-workers (Lagerstedt, J. O.; Voss, J. C.; Wieslander, Å.; Persson, B. L. *FEBS Lett.* **2004**, 578, 262-268.)

Atomic Distances

P_i - Lys[492] & P_i - Asp[178]/Asp[178]-H

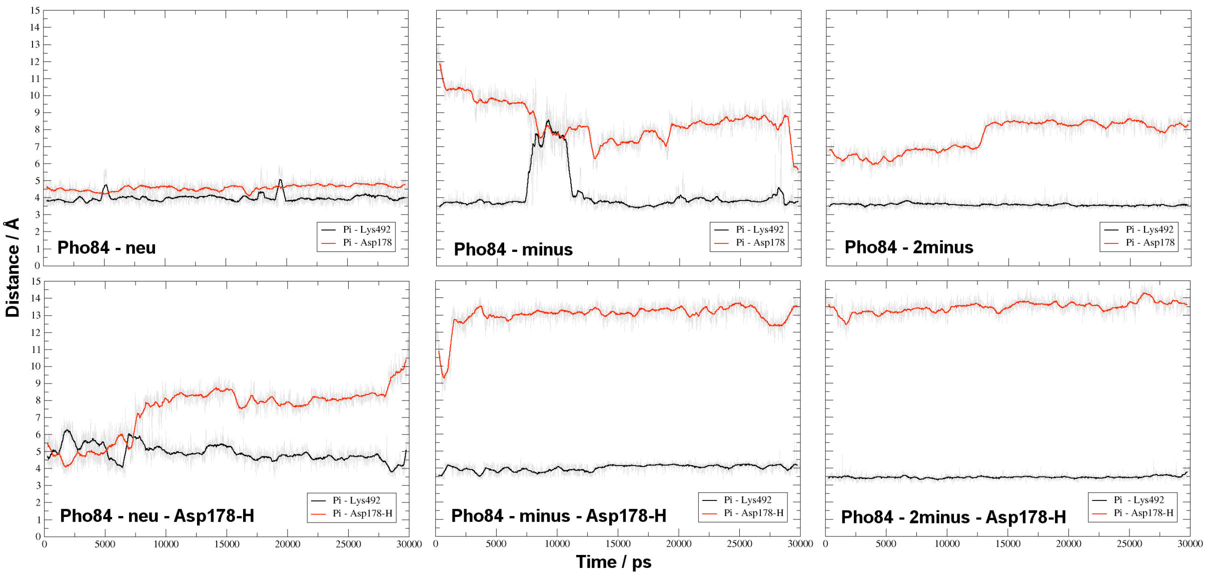

Figure S18. MD equilibration data for 30 ns simulations showing the variation in the atomic distances between the central phosphorus atom of P_i and the side chain nitrogen atom of Lys[492] and between the central atom of P_i and the the side chain carboxylic acid carbon of Asp[178] or of Asp[178]-H during equilibration (in gray). Data are also presented as a running average of 50 data points (in red). For a detailed description of each system, refer to the figure legends for Figures S1-S8.

Asp[178]/Asp[178]-H - Asp[76]

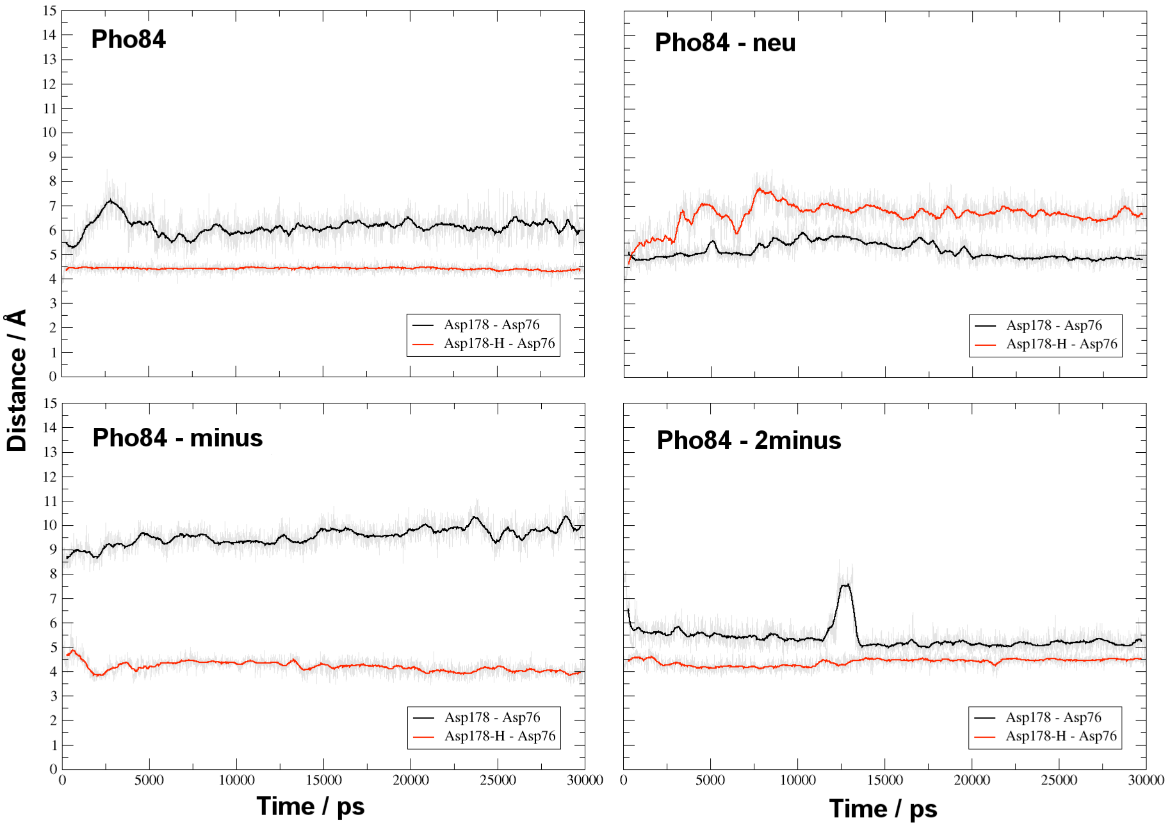

Figure S19. MD equilibration data extracted from 30 ns simulations showing the variation in the atomic distances between the the side chain carboxylic acid carbons of Asp[178] or Asp[178]-H and the side chain carboxylic acid carbon of Asp[76] during equilibration (in gray) Data are also presented as a running average of 50 data points (in red). For a detailed description of each system, refer to the figure legends for Figures S1-S8.

P_i - Tyr[179]

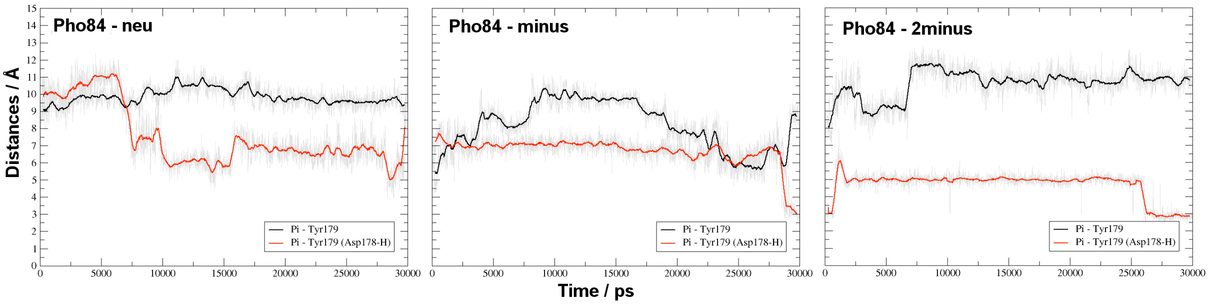

Figure S20. MD equilibration data extracted from 30 ns simulations showing variation in the atomic distances between the central phosphorus atom of P_i and the side chain hydroxyl group hydrogen atom of Tyr[179] during equilibration (in gray). Data are also presented as a running average of 50 data points (in red). For a detailed description of each system, refer to the figure legends for Figures S1-S8.

SMD Simulation Data

Force Profiles:

Pho84 – neu / Pho84 – minus – Asp178-H / Pho84 – minus

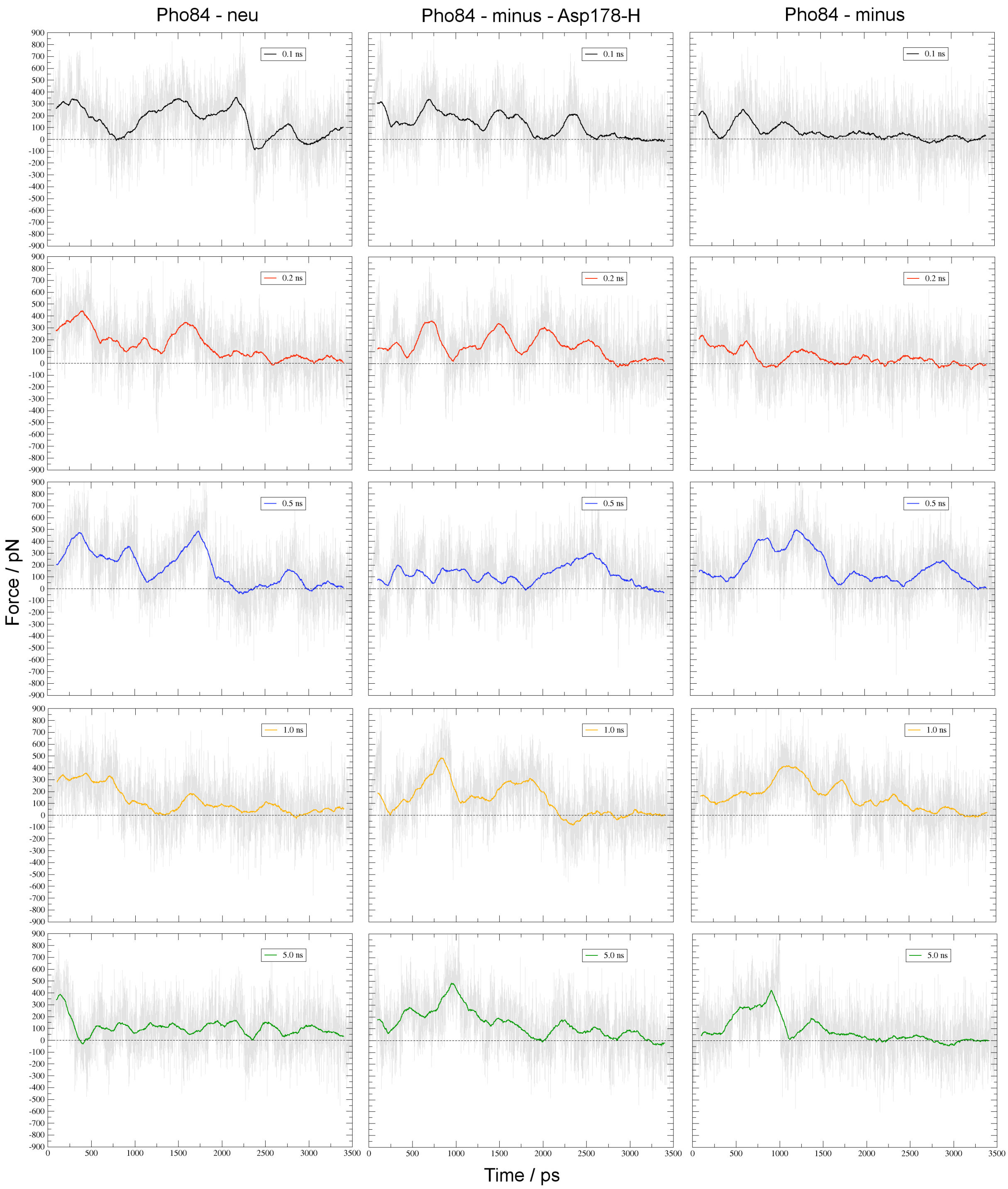

Figure S21. Force profiles derived from simulations of the cytosolic release of various protonation states of P_i from Pho84: H_3PO_4 (Pho84 – neu); $H_2PO_4^-$,and protonated Asp^{178}-H (Pho84 – minus – Asp178-H); or $H_2PO_4^-$ (Pho84 – minus). Various equlibration times were implemented (black line: 0.1 ns, red line: 0.2 ns, blue line: 0.5 ns, orange line: 1.0 ns, and green line: 5.0 ns) prior to increasing the distance between the central phosphorus atom of P_i and the side chain nitrogen atom of Lys^{492} for 3.5 ns using constant velocity SMD. The data is depicted as a running average of 200 data points.

Pho84 – 2minus

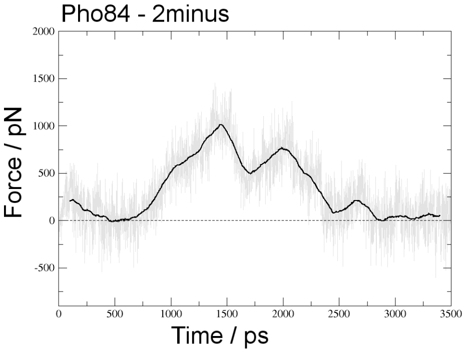

Figure S22. The force profile derived from a simulation of the cytosolic release of HPO_4^{2-} from Pho84 (Pho84 – 2minus). A 0.1 ns equilibration time was implemented prior to increasing the distance between the central phosphorus atom of P_i and the side chain nitrogen atom of Lys[492] for 3.5 ns using constant velocity SMD. The data is depicted as a running average of 200 data points (black line).

Work Profiles:

Pho84 – neu / Pho84 – minus – Asp178-H / Pho84 - minus

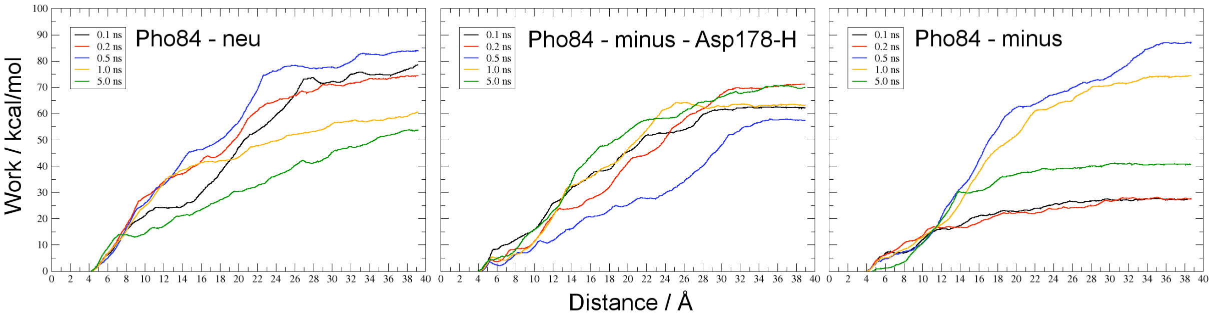

Figure S23. Work profiles derived from simulations of the cytosolic release of different protonation states of P_i from Pho84: H_3PO_4 (Pho84 – neu); $H_2PO_4^-$ and a protonated Asp178-H (Pho84 – minus – Asp178-H); or $H_2PO_4^-$ (Pho84 – minus). Various equilibration times were implemented (black line: 0.1 ns, red line: 0.2 ns, blue line: 0.5 ns, orange line: 1.0 ns, and green line:5.0 ns) prior to increasing the distance between the central phosphorus atom of P_i and the side chain nitrogen atom of Lys[492] for 3.5 ns using constant velocity SMD.

Pho84 – 2minus

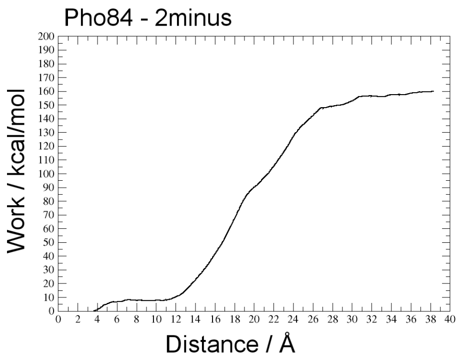

Figure S24. Work profile derived from a simulation of the cytosolic release of HPO_4^{2-} from Pho84 (Pho84 – 2minus). A 0.1 ns equlibration time was implemented prior to increasing the distance between the central phosphorus atom of P_i and the side chain nitrogen atom of Lys[492] for 3.5 ns using constant velocity SMD.

Pho84 Residue-P_i Hydrogen Bonding:

Pho84 – neu

Table S2. Hydrogen bonding (using 3.0Å and 20° interaction cut-offs) as a percentage of the total simulation time for the cytosolic release of H_3PO_4 from Pho84 (Pho84 – neu) using a 0.1, 0.2, 0.5, 1.0, or 5.0 ns equilibration stage prior to increasing the distance between the central phosphorus atom of P_i and the side chain nitrogen atom of Lys[492] for 3.5 ns using constant velocity steered molecular dynamics. Only extracted values ≥1% are presented.

		**H-bonding (%)**				
**Residue**	**Fragment[a]**	**0.1 ns**	**0.2 ns**	**0.5 ns**	**1.0 ns**	**5.0 ns**
Gln119	H-II	3.0	7.0	5.3	3.9	4.7
Asp127	L-II		3.9			
Gly176	H-IV	6.1	5.9	7.9	6.1	1.6
Asp178	H-IV	4.7	3.4	3.6	3.6	
Ser183	H-IV					2.1
Leu286	L-VI		3.7			
Gln291	L-VI					1.0
Hie300	L-VI	3.9				
Asp306	L-VI				2.4	
Gly311	L-VI			4.7		
Glu313	L-VI	2.7	1.3	6.0		6.0
Arg314	L-VI	1.4				1.0
Ala315	L-VI					1.6
Ser316	L-VI	1.0				
Thr317	L-VI					5.7
Ala318	L-VI	2.4				7.3
Glu320	L-VI	1.9				
Ser321	L-VI	2.3				4.0
Leu322	L-VI		1.3			
Pro326	L-VI			2.4		
Lys328	L-VI	5.1				4.9
Ser330	L-VI	4.1	3.6	2,0		1.3
Phe331	L-VI			2.6		
Lys332	L-VI					
Asp333	L-VI			7.1	1.6	
Phe334	L-VI			2.1	3.7	
His337	L-VI	6.3				1.9
Gln340	L-VI	2.0				
Arg478	L-X				2.0	
Tyr479	H-XI			1.6		
Glu551	H-XII				1.3	

[a] Derived from the topological model of Pho84 previously presented by Lagerstedt et al. (Lagerstedt, J. O.; Voss, J. C.; Wieslander, Å.; Persson, B. L. *FEBS Lett.* **2004**, 578, 262-268.)

Pho84 – minus – Asp178-H

Table S3. Hydrogen bonding (using 3.0 Å and 20° interaction cut-offs) as a percentage of the total simulation time for the cytosolic release of $H_2PO_4^-$ from Pho84 with protonated Asp^{178} (Pho84 – minus – Asp178-H) using a 0.1, 0.2, 0.5, 1.0, or 5.0 ns equilibration stage prior to increasing the distance between the central phosphorus atom of P_i and the side chain nitrogen atom of Lys^{492} for 3.5 ns using constant velocity steered molecular dynamics. Only extracted values ≥1% are presented.

		H-bonding (%)				
**Residue**	**Fragment[a]**	**0.1 ns**	**0.2 ns**	**0.5 ns**	**1.0 ns**	**5.0 ns**
Thr115	H-II					2.0
Gln119	H-II	13.7	13.0	9.7	10.6	7.9
Arg131	H-III				2.4	
Lys132	H-III				2.4	
Tyr179	H-IV	15.6	17.1	3.0	19.9	5.0
Pro180	H-IV		1.6			
Ser183	H-IV		13.0	6.3	2.3	
Ser187	L-IV			4.4		
Thr192	L-IV	2.1				
Trp194	L-IV	4.3				
Arg275	H-VI				3.4	
Lys283	L-VI				4.4	
Glu285	L-VI	1.1	1.1		1.7	
Leu286	L-VI		2.0	8.6		
Ala287	L-VI		1.1			
Ala288	L-VI			1.0		
Ile299	L-VI					1.0
Glu313	L-VI					1.7
Arg314	L-VI	6.3			2.6	15.6
Thr317	L-VI					1.6
Ser330	L-VI				2.0	
Lys332	L-VI	4.6	15.4	10.3	8.6	
Asp333	L-VI	2.6	5.9	7.3		
Trp341	L-VI					3.9
Lys342	L-VI					1.0
Arg478	L-X	10.4		5.0		
Ser481	H-XI	13.7				
His484	H-XI	4.7	4.0	3.1	2.9	
Lys492	H-XI	2.1	1.0	1.4	1.1	1.0

[a] Derived from the topological model of Pho84 previously presented by Lagerstedt et al. (Lagerstedt, J. O.; Voss, J. C.; Wieslander, Å.; Persson, B. L. *FEBS Lett.* **2004**, 578, 262-268.)

Pho84 – minus

Table S4. Hydrogen bonding (using 3.0 Å and 20° interaction cut-offs) as a percentage of the total simulation time for the cytosolic release of $H_2PO_4^-$ from Pho84 (Pho84 – minus) using a 0.1, 0.2, 0.5, 1.0, or 5.0 ns equilibration stage prior to increasing the distance between the central phosphorus atom of P_i and the side chain nitrogen atom of Lys^{492} for 3.5 ns using constant velocity steered molecular dynamics. Only extracted values ≥1% are presented.

		H-bonding (%)				
**Residue**	**Fragment[a]**	**0.1 ns**	**0.2 ns**	**0.5 ns**	**1.0 ns**	**5.0 ns**
Ala69	H-I				1.1	
Gly72	H-I				2.4	
Gln119	H-II			3.1		
Arg131	H-III				1.6	
Tyr135	H-III				1.0	
Ile175	H-IV	3.3	1.7	1.7	3.3	
Asp178	H-IV		1.3	1.6	10.7	
Tyr179	H-IV	3.4	2.6			
Ser182	H-IV	1.1	2.3		6.3	
Ser183	H-IV	5.1	3.6			
Ile185	H-IV				2.7	
Ser187	L-IV		2.3			
Arg195	L-IV					8.6
Ser273	H-VI				1.3	
Asp279	L-VI				8.6	
Asn281	L-VI				2.9	
Lys283	L-VI				2.1	
Gln291	L-VI			10.9		
Glu292	L-VI			4.6		
Gln293	L-VI			3.3		
Asp306	L-VI		1.0			
Ser316	L-VI			11.4		
Glu320	L-VI		1.1			
Ser321	L-VI		1.1			
Lys332	L-VI	3.0	2.1	1.1		
Cys335	L-VI		1.0			
Arg336	L-VI	6.9	5.3			8.7
Thr477	L-X	2.6				1.3
Ser481	H-XI	12.1				6.1
Ala488	H-XI					1.9
Lys492	H-XI	1.1	1.3		2.4	
Glu551	H-XII					3.0

[a] Derived from the topological model of Pho84 previously presented by Lagerstedt et al. (Lagerstedt, J. O.; Voss, J. C.; Wieslander, Å.; Persson, B. L. *FEBS Lett.* **2004**, 578, 262-268.)

Pho84 – 2minus

Table S5. Hydrogen bonding (using 3.0 Å and 20° interaction cut-offs) as a percentage of the total simulation time for the cytosolic release of HPO_4^{2-} from Pho84 (Pho84 – 2minus) using a 0.1 ns equilibration stage prior to increasing the distance between the central phosphorus atom of P_i and the side chain nitrogen atom of Lys492 for 3.5 ns using constant velocity steered molecular dynamics. Only extracted values ≥1% are presented.

**Residue**	**Fragment**[a]	**H-bonding (%)**
Thr115	H-II	29.4
Gln119	H-II	14.7
Thr124	H-II	13.3
Lys297	L-VI	2.4
Arg314	L-VI	39.9
Thr317	L-VI	13.6
Lys332	L-VI	15.9

[a] Derived from the topological model of Pho84 previously presented by Lagerstedt et al. (Lagerstedt, J. O.; Voss, J. C.; Wieslander, Å.; Persson, B. L. *FEBS Lett.* **2004**, 578, 262-268.)

Pho84 Residue-P_i Hydrogen Bond Formation and Disruption Lifetime:

Pho84 – neu

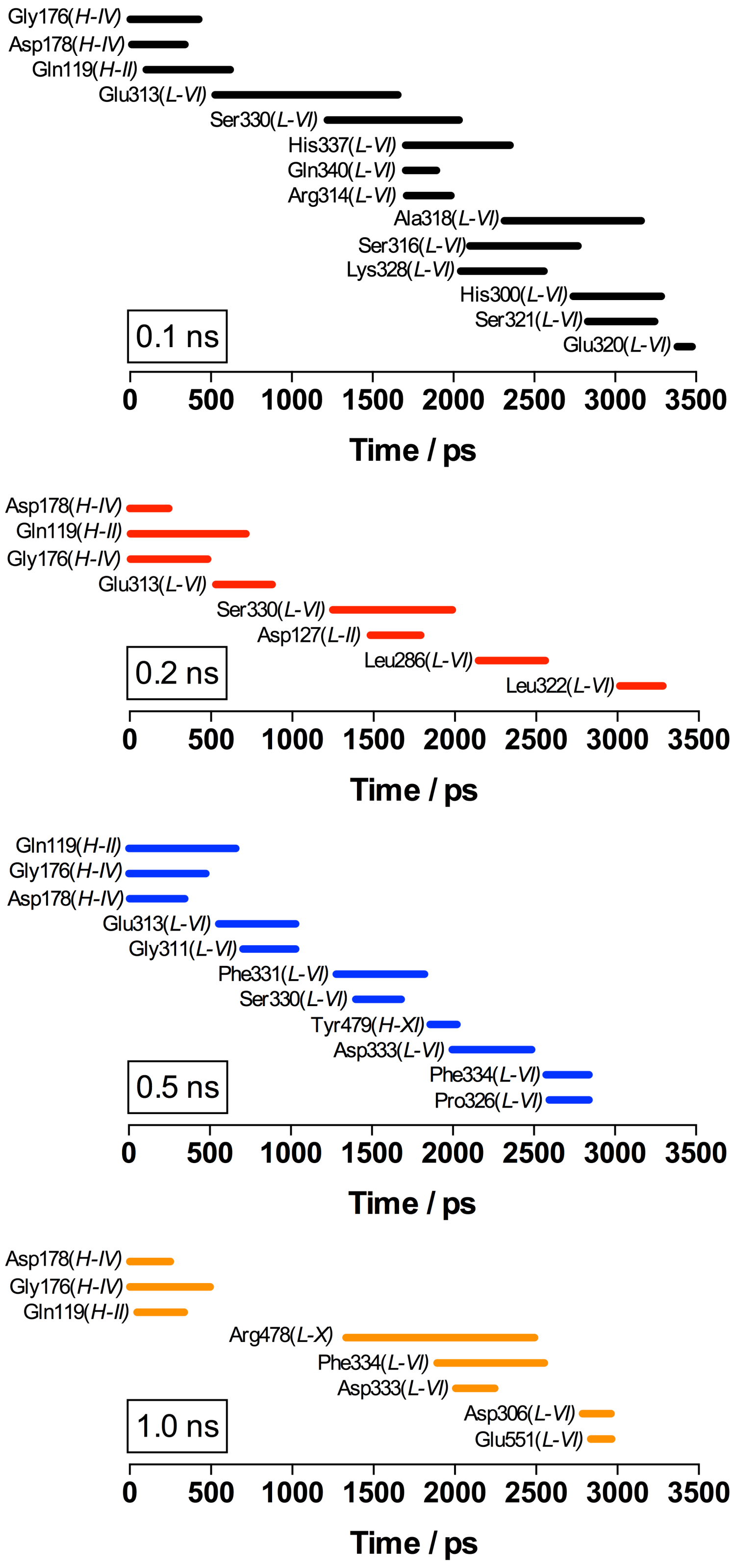

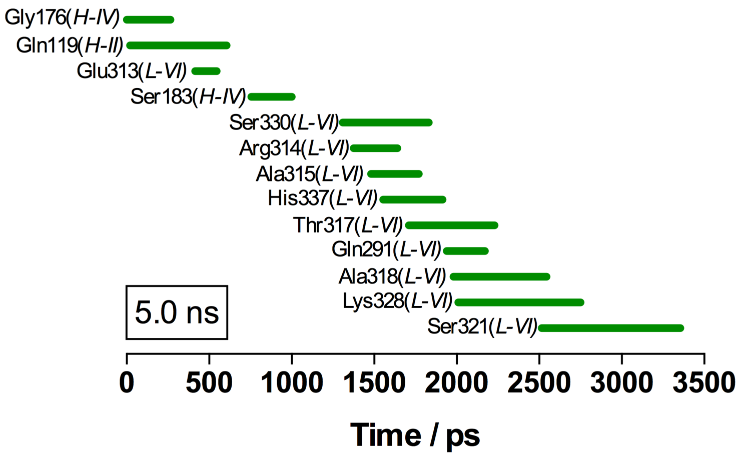

Figure S25. Hydrogen bond formation and disruption lifetime (using 3.0 Å and 20° interaction cut-offs) for the cytosolic release of H_3PO_4 from Pho84 (Pho84 – neu) using 0.1, 0.2, 0.5, 1.0, or 5.0 ns equilibration stage prior to increasing the distance between the central phosphorus atom of P_i and the side chain nitrogen atom of Lys[492] for 3.5 ns using constant velocity SMD.

Pho84 – minus – Asp178-H

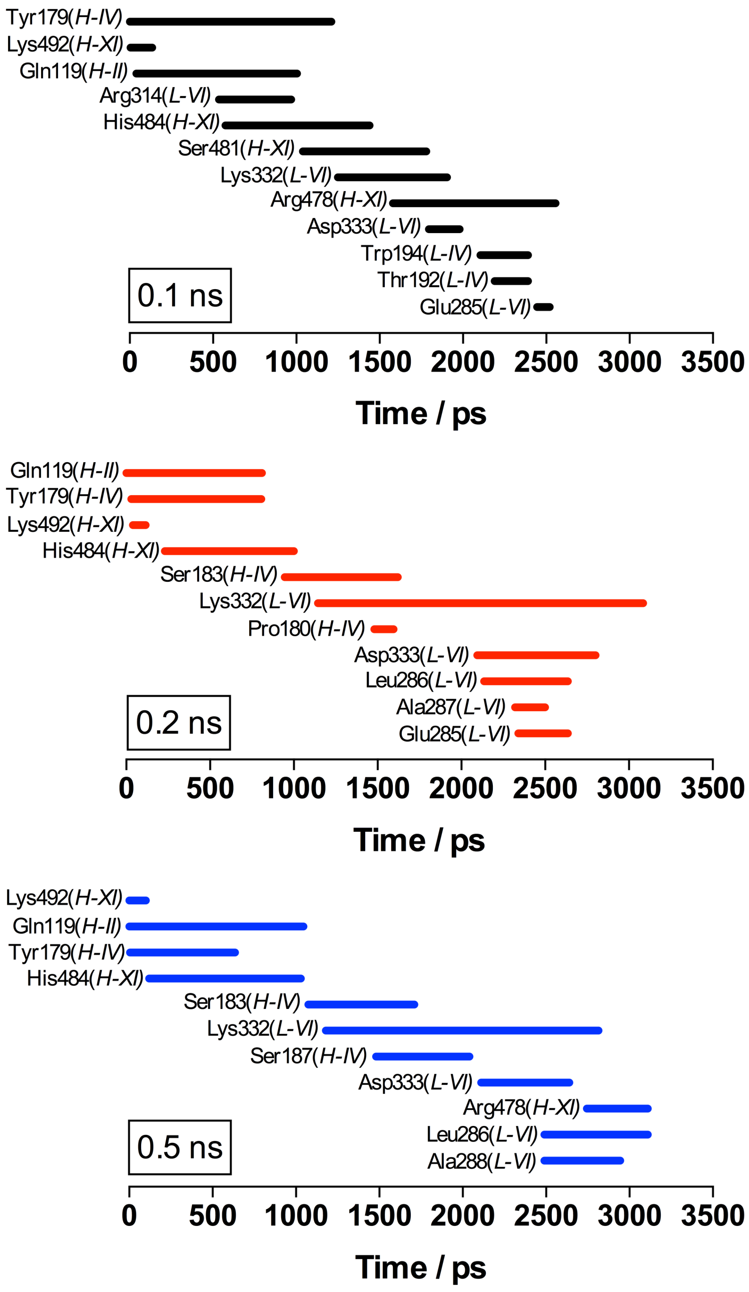

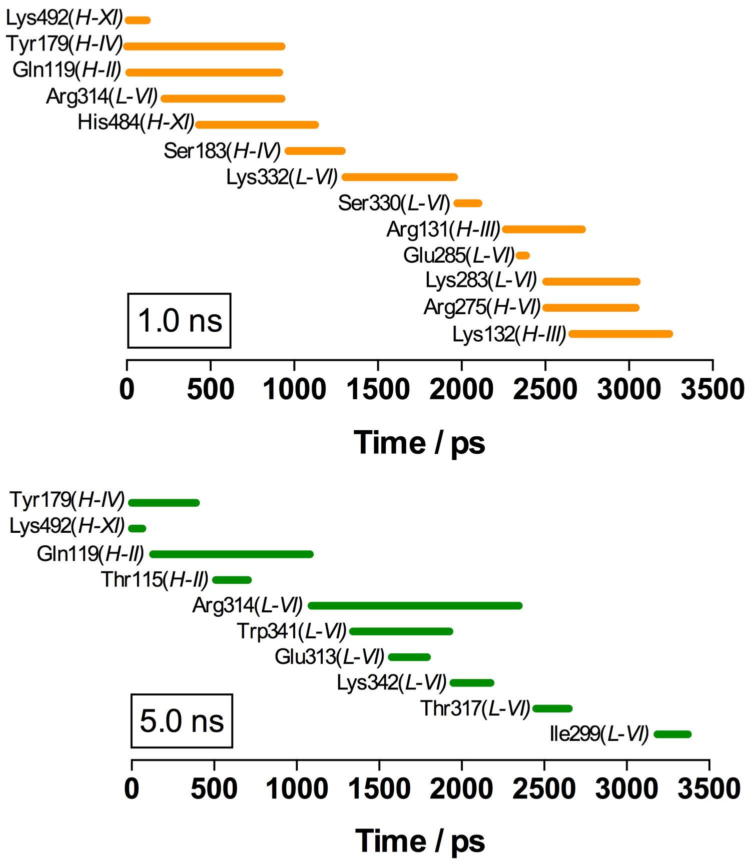

Figure S26. Hydrogen bond formation and disruption lifetime (using 3.0 Å and 20° interaction cut-offs) for the cytosolic release of $H_2PO_4^-$ from Pho84 with protonated Asp[178] (Pho84 – minus – Asp178-H) using a 0.1, 0.2, 0.5, 1.0, or 5.0 ns equilibration stage prior to increasing the distance between the central phosphorus atom of P_i and the side chain nitrogen atom of Lys[492] for 3.5 ns using constant velocity SMD.

Pho84 – minus

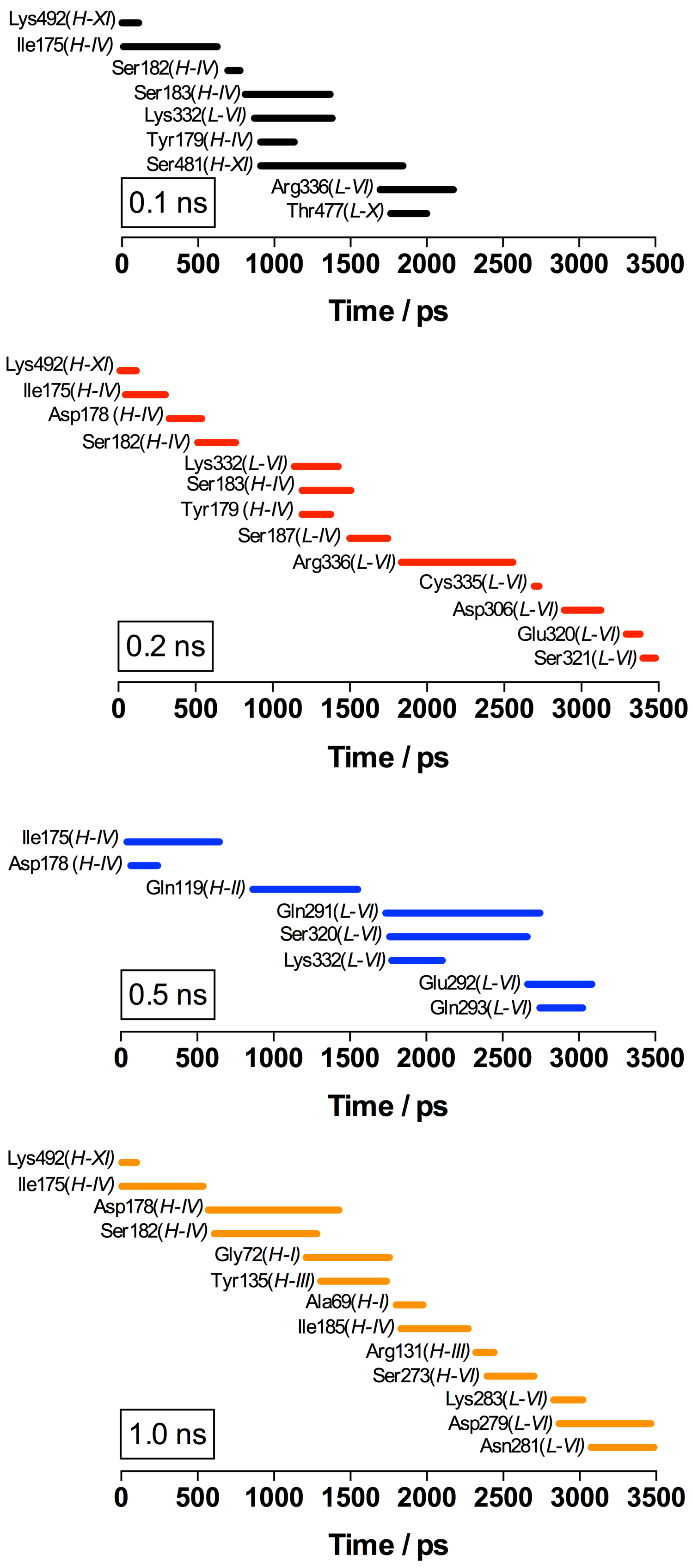
Lys492(H-XI)
Ile175(H-IV)
Ser182(H-IV)
Ser183(H-IV)
Lys332(L-VI)
Tyr179(H-IV)
Ser481(H-XI)
Arg336(L-VI)
Thr477(L-X)
0.1 ns
0 500 1000 1500 2000 2500 3000 3500
Time / ps
Lys492(H-XI)
Ile175(H-IV)
Asp178 (H-IV)
Ser182(H-IV)
Lys332(L-VI)
Ser183(H-IV)
Tyr179 (H-IV)
Ser187(L-IV)
Arg336(L-VI)
Cys335(L-VI)
Asp306(L-VI)
Glu320(L-VI)
Ser321(L-VI)
0.2 ns
0 500 1000 1500 2000 2500 3000 3500
Time / ps
Ile175(H-IV)
Asp178 (H-IV)
Gln119(H-II)
Gln291(L-VI)
Ser320(L-VI)
Lys332(L-VI)
Glu292(L-VI)
Gln293(L-VI)
0.5 ns
0 500 1000 1500 2000 2500 3000 3500
Time / ps
Lys492(H-XI)
Ile175(H-IV)
Asp178(H-IV)
Ser182(H-IV)
Gly72(H-I)
Tyr135(H-III)
Ala69(H-I)
Ile185(H-IV)
Arg131(H-III)
Ser273(H-VI)
Lys283(L-VI)
Asp279(L-VI)
Asn281(L-VI)
1.0 ns
0 500 1000 1500 2000 2500 3000 3500
Time / ps

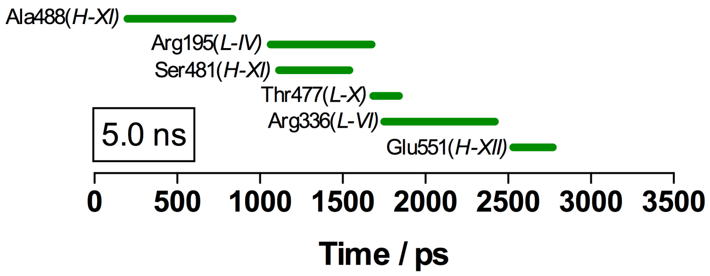

Figure S27. Hydrogen bond formation and disruption lifetime (using 3.0 Å and 20° interaction cut-offs) for the cytosolic release of $H_2PO_4^-$ from Pho84 (Pho84 – minus) using a 0.1, 0.2, 0.5, 1.0, or 5.0 ns equilibration stage prior to increasing the distance between the central phosphorus atom of P_i and the side chain nitrogen atom of Lys^{492} for 3.5 ns using constant velocity SMD.

Pho84 – 2minus

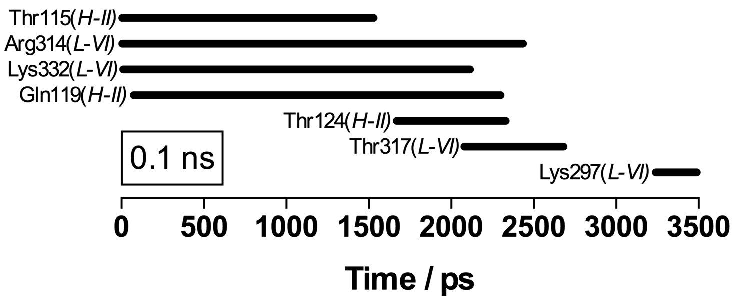

Figure S28. Hydrogen bond formation and disruption lifetime (using 3.0Å and 20° interaction cut-offs) for the cytosolic release of HPO_4^{2-} from Pho84 (Pho84 – 2minus) using a 0.1 ns equilibration stage prior to increasing the distance between the central phosphorus atom of P_i and the side chain nitrogen atom of Lys^{492} for 3.5 ns using constant velocity SMD.

Water-P_i Hydrogen Bonding:

Pho84 – neu / Pho84 – minus – Asp178-H / Pho84 – minus

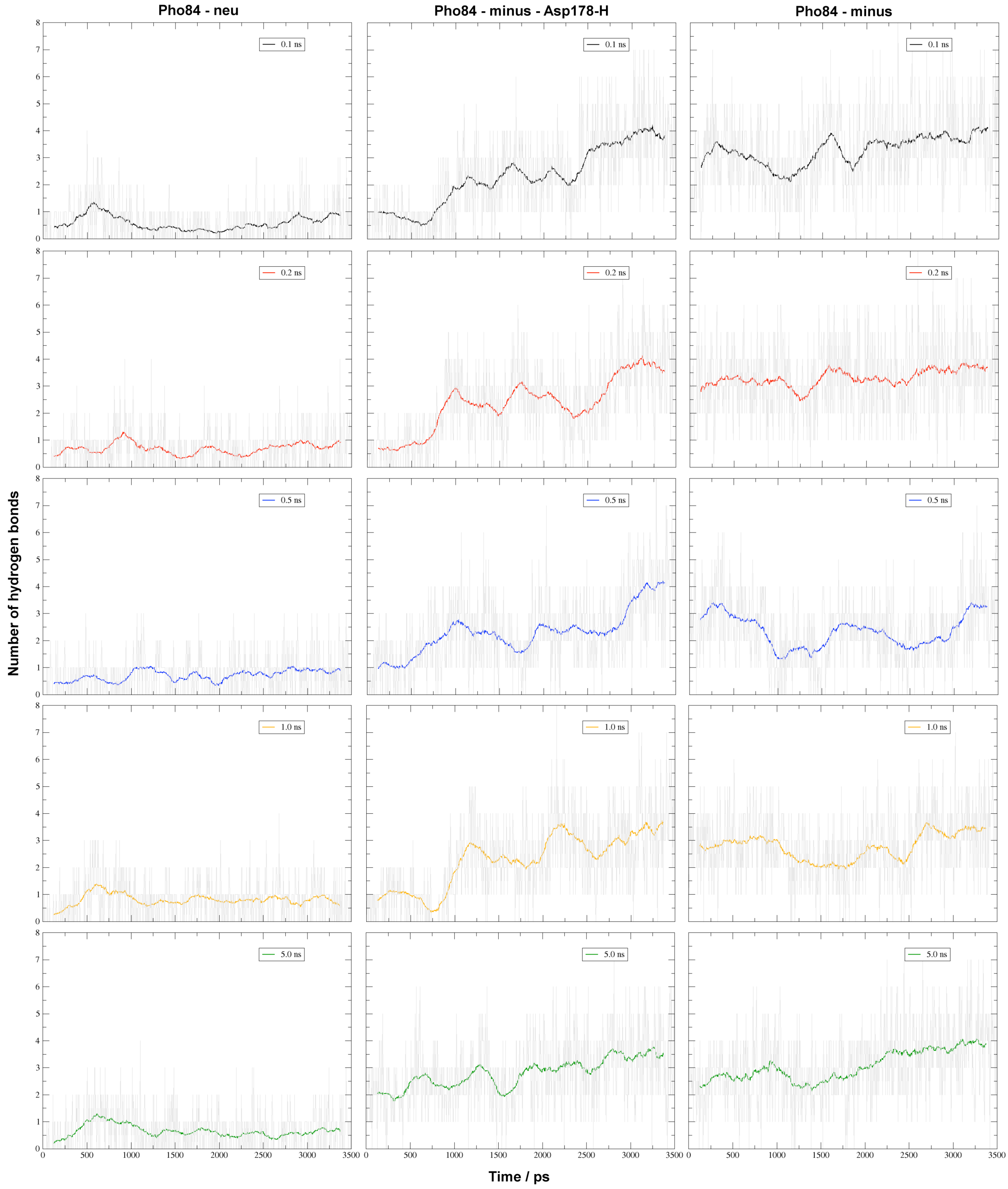

Figure S29. Water-P_i hydrogen bonding profiles (using 3.0Å and 20° interaction cut-offs) derived from simulations of the cytosolic release of various protonation states of P_i from Pho84: H_3PO_4 (Pho84 – neu); $H_2PO_4^-$ and protonated Asp[178]-H (Pho84 – minus – Asp178-H); or $H_2PO_4^-$ (Pho84 – minus). The various equilibration times were implemented (black line: 0.1 ns, red line: 0.2 ns, blue line: 0.5 ns, orange line: 1.0 ns, and green line: 5.0 ns) prior to increasing the distance between the central phosphorus atom of P_i and the side chain nitrogen atom of Lys[492] for 3.5 ns using constant velocity SMD. Data are depicted as a running average of 50 data points.

Pho84 – 2minus

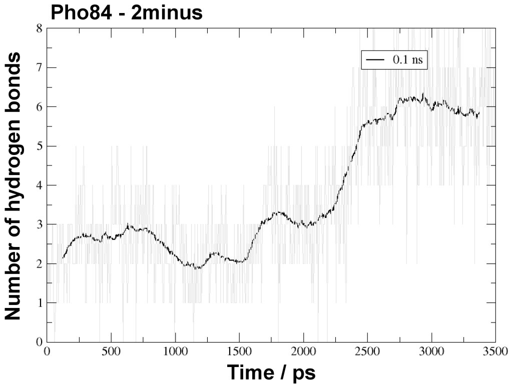

Figure S30. A water-P_i hydrogen bonding profile (using 3.0 Å and 20° interaction cut-offs) derived from a simulation of the cytosolic release of HPO_4^{2-} from Pho84 (Pho84 – 2minus). A 0.1 ns equlibration time was implemented prior to increasing the distance between the central phosphorus atom of P_i and the side chain nitrogen atom of Lys^{492} for 3.5 ns using constant velocity SMD. Data are depicted as a running average of 50 data points (black line).

Release Routes:

Pho84 – neu

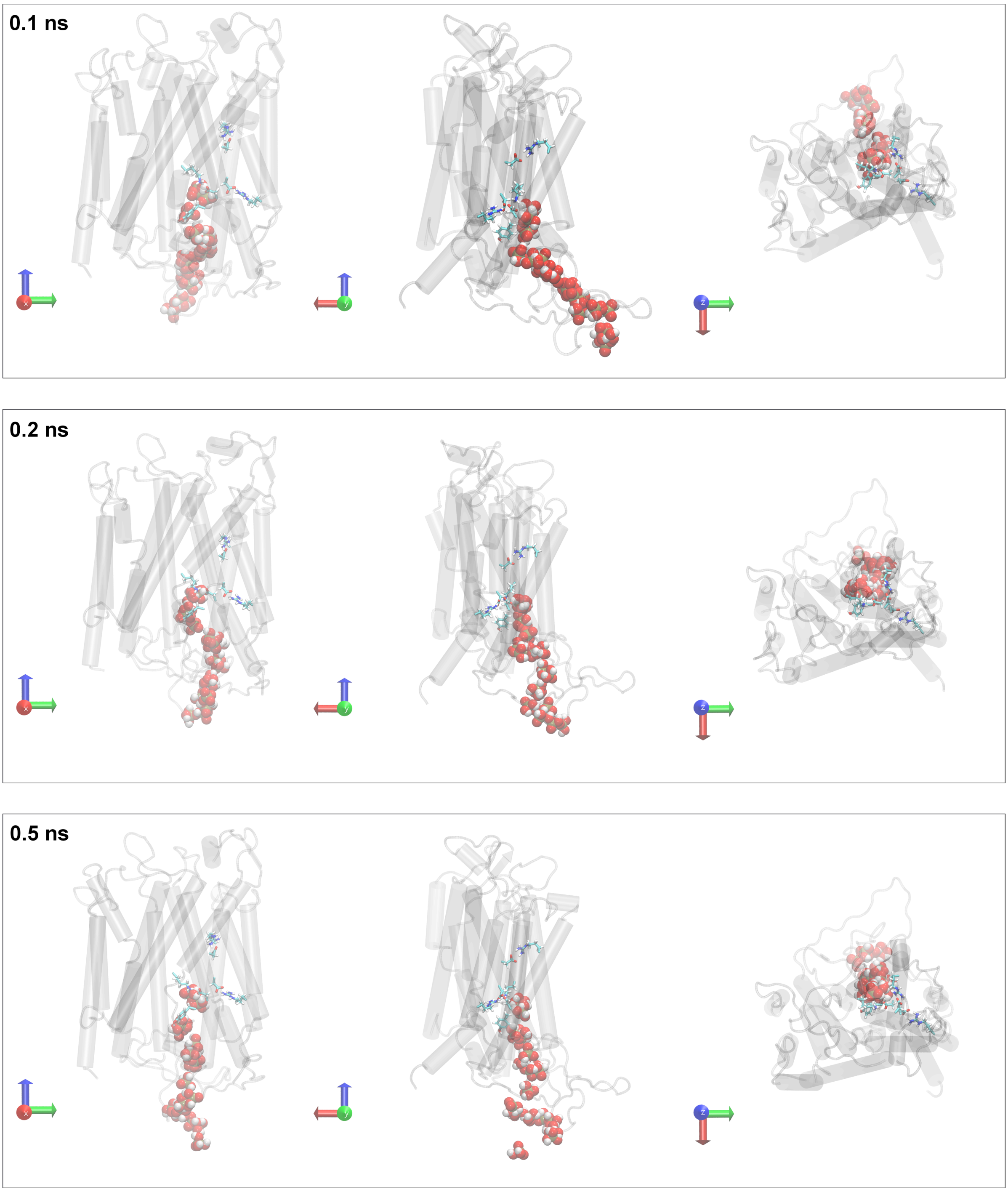

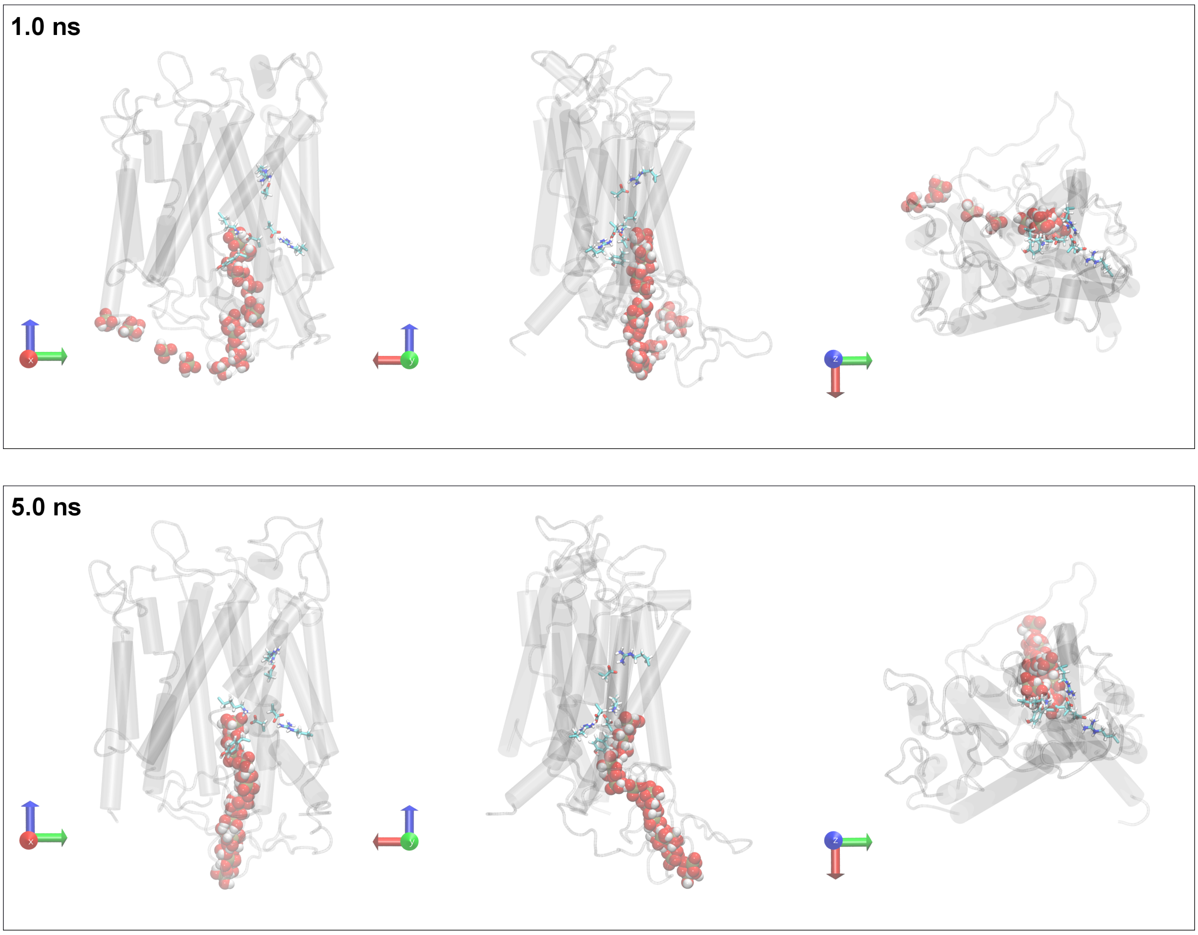

Figure S31. Snapshots derived from SMD simulations of Pho84 obtained prior to 3.5 ns of simulation and multiple structures with H_3PO_4 (Pho84 – neu) extracted every 0.175 ns (van der Waal volume representations). SMD simulations were performed after initially restraining the Lys^{492}-P$_i$ distance for 0.1, 0.2, 0.5, 1.0, or 5.0 ns. Residues shown (licorice representations) are those that are proposed to be important for binding P$_i$ (Lys^{492}, Asp^{178}, Tyr^{179}) and those that are involved in proton transfer (Asp^{76}, Asp^{79}, Asp^{178}, Arg^{168}, and Arg^{267}). The coordinate axes are shown in green (x-axis), red (y-axis), and blue (z-axis).

Pho84 – minus – Asp178-H

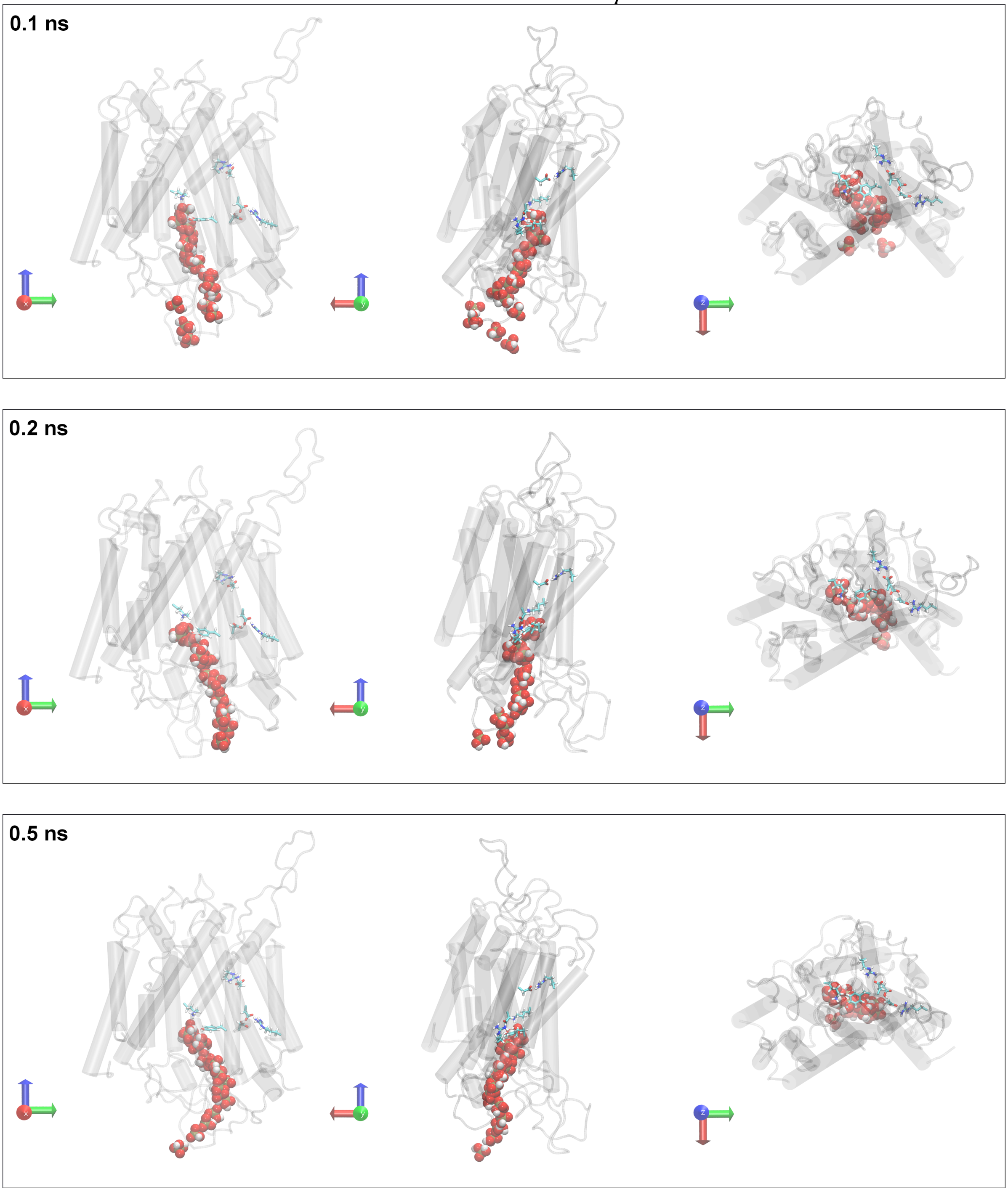

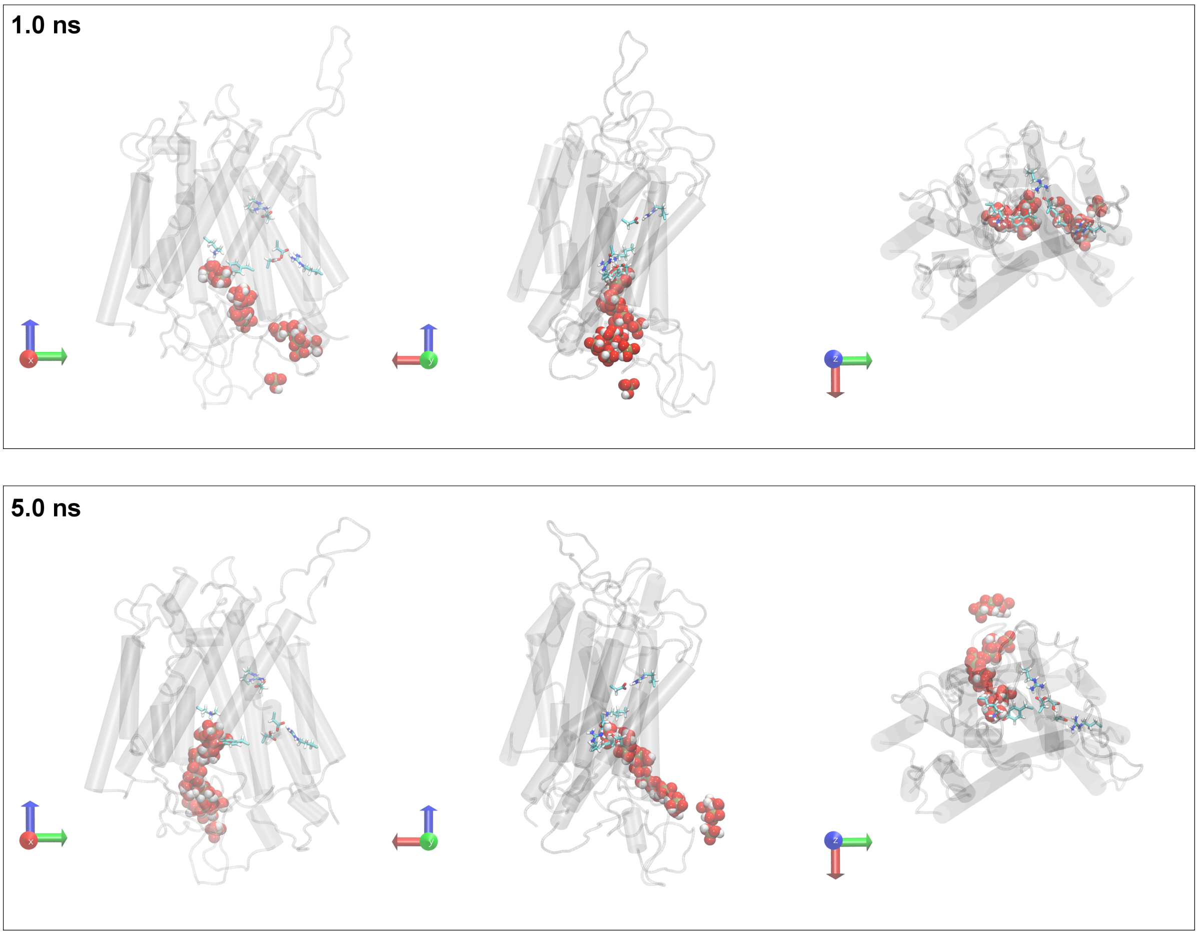

Figure S32. Snapshots derived from SMD simulations of Pho84 obtained prior to 3.5 ns of simulation and multiple structures with $H_2PO_4^-$ and protonated Asp[178]-H (Pho84 – minus – Asp178-H) extracted every 0.175 ns (h van der Waal volume representations). SMD simulations were performed after initially restraining the Lys[492]-P_i distance for 0.1, 0.2, 0.5, 1.0, or 5.0 ns. Residues shown (licorice representations) are those proposed to be important for binding P_i (Lys[492], Asp[178], Tyr[179]) and those that are involved in proton transfer (Asp[76], Asp[79], Asp[178], Arg[168], and Arg[267]). The coordinate axes are shown in green (x-axis), red (y-axis), and blue (z-axis).

Pho84 – minus

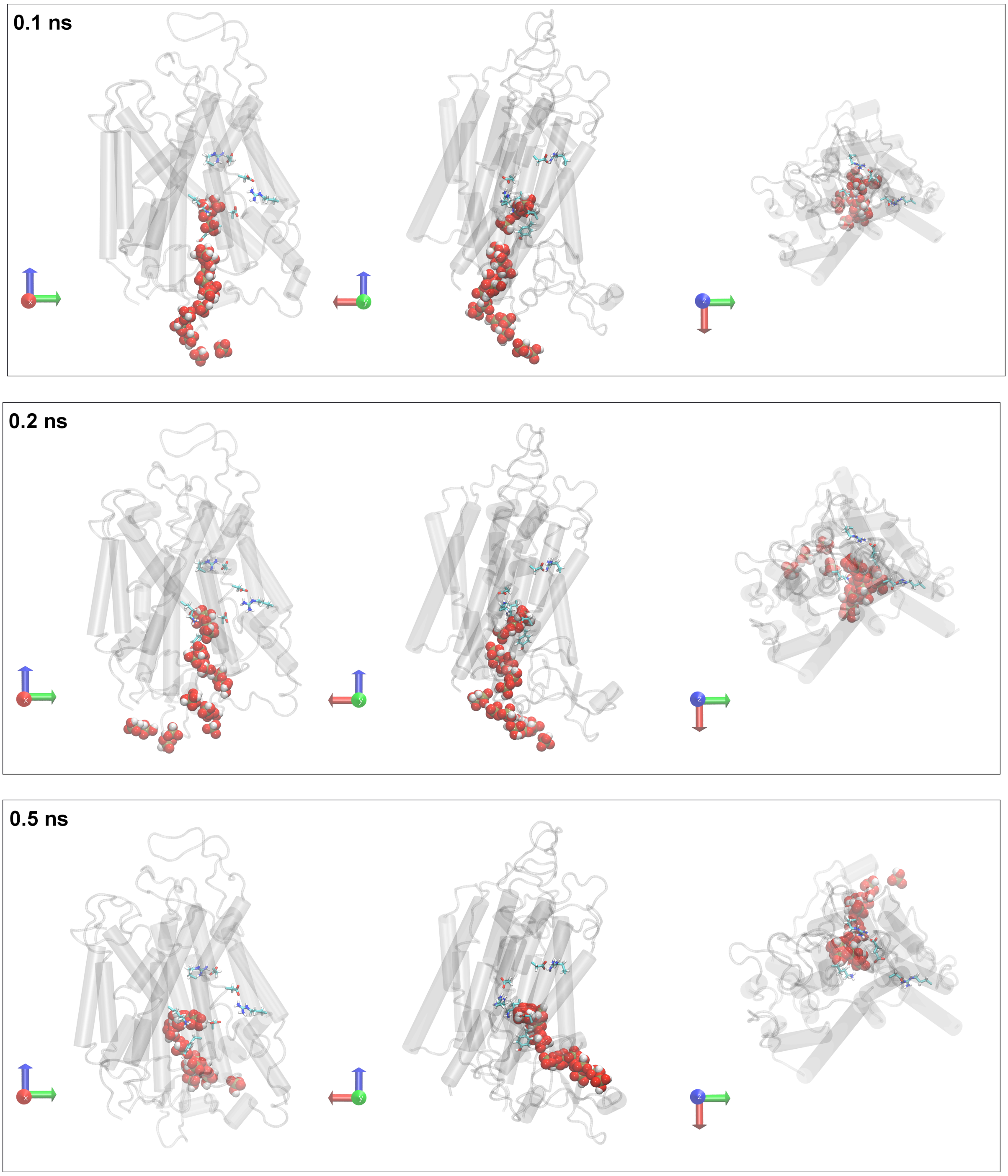

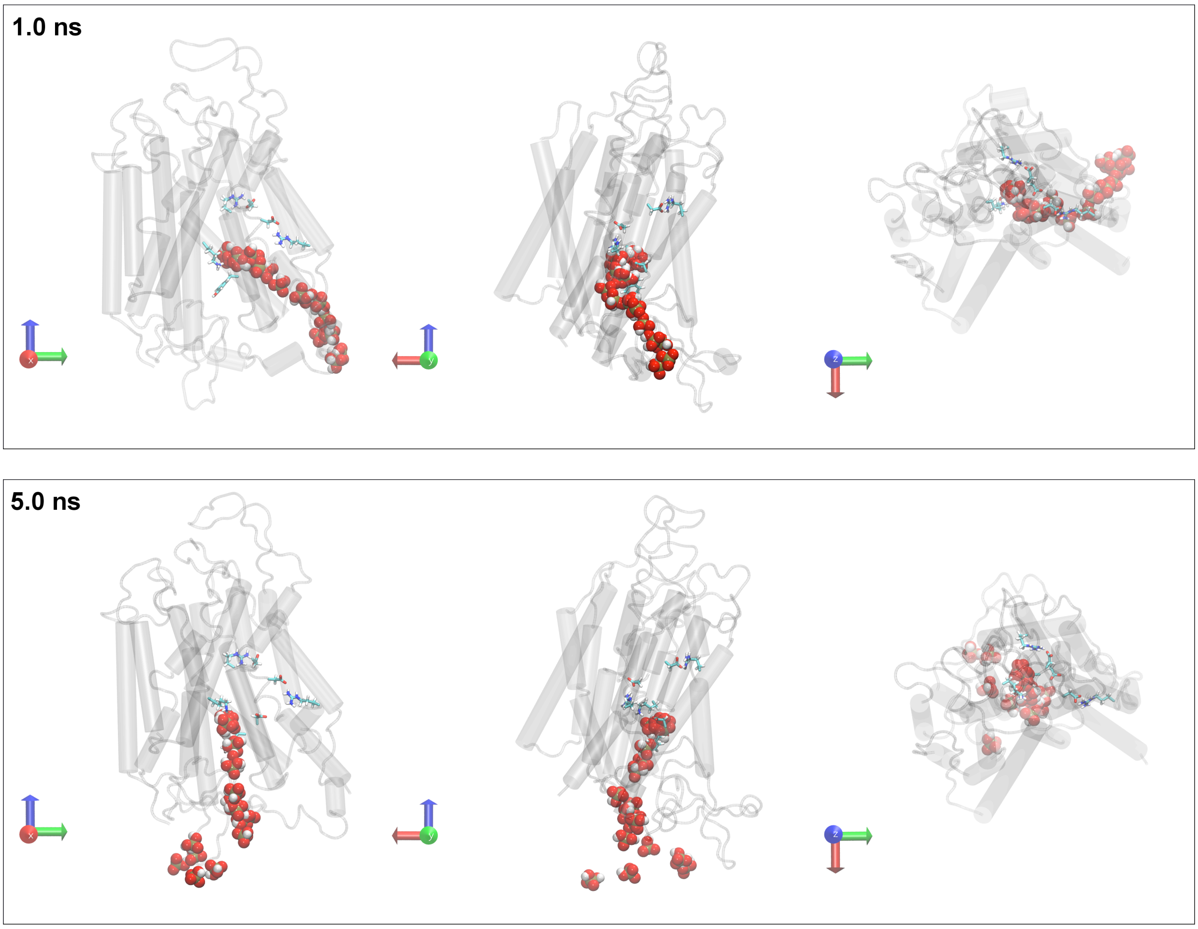

Figure S33. Snapshots derived from SMD simulations of Pho84 obtained prior to 3.5 ns of simulation and multiple structures with $H_2PO_4^-$ (Pho84 – minus) extracted every 0.175 ns (van der Waal volume representations). SMD simulations were performed after initially restraining the Lys^{492}-P_i distance for 0.1, 0.2, 0.5, 1.0, or 5.0 ns. Residues shown (licorice representations) are those proposed to be important for binding P_i (Lys^{492}, Asp^{178}, Tyr^{179}) and those that are involved in proton transfer (Asp^{76}, Asp^{79}, Asp^{178}, Arg^{168}, and Arg^{267}). The coordinate axes are shown in green (x-axis), red (y-axis), and blue (z-axis).

Pho84 – 2minus

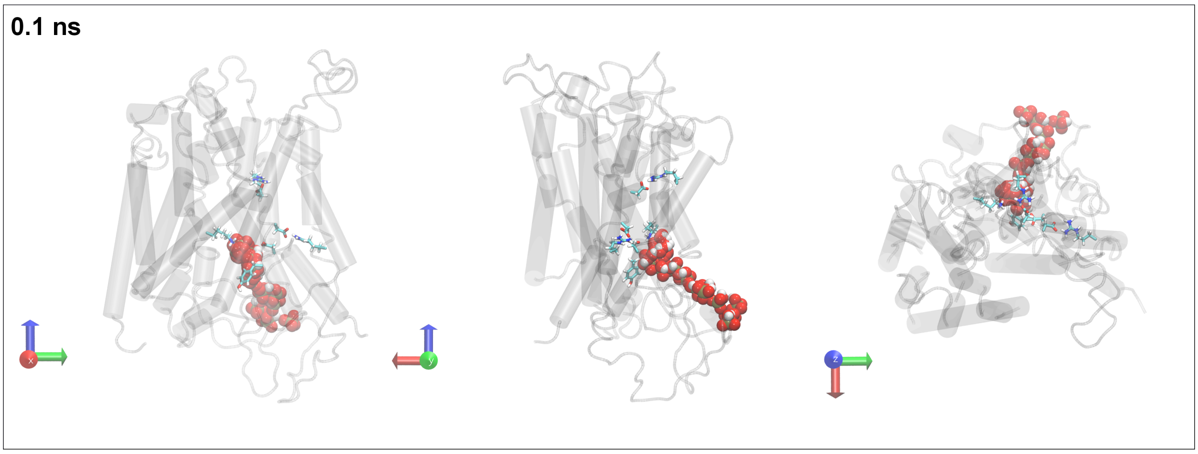

Figure S34. Snapshots derived from SMD simulations of Pho84 obtained prior to 3.5 ns of simulation and multiple structures with HPO_4^{2-} (Pho84 – 2minus) extracted every 0.175 ns (van der Waal volume representations). SMD simulations were performed after initially restraining the Lys^{492}-P_i distance for 0.1, 0.2, 0.5, 1.0, or 5.0 ns. Residues shown (licorice representations) are those proposed to be important for binding P_i (Lys^{492}, Asp^{178}, Tyr^{179}) or those that are involved in the proton transfer system (Asp^{76}, Asp^{79}, Asp^{178}, Arg^{168}, and Arg^{267}). The coordinate axes are shown in green (x-axis), red (y-axis), and blue (z-axis).
